# KChIP2 genotype dependence of transient outward current (I_to_) properties in cardiomyocytes isolated from male and female mice

**DOI:** 10.1371/journal.pone.0171213

**Published:** 2017-01-31

**Authors:** Lara Waldschmidt, Vera Junkereit, Robert Bähring

**Affiliations:** Institut für Zelluläre und Integrative Physiologie, Zentrum für Experimentelle Medizin, Universitätsklinikum Hamburg-Eppendorf, Hamburg, Germany; University of Texas Health Science Center, UNITED STATES

## Abstract

The transient outward current (I_to_) in cardiomyocytes is largely mediated by Kv4 channels associated with Kv Channel Interacting Protein 2 (KChIP2). A knockout model has documented the critical role of KChIP2 in I_to_ expression. The present study was conducted to characterize in both sexes the dependence of I_to_ properties, including current magnitude, inactivation kinetics, recovery from inactivation and voltage dependence of inactivation, on the number of functional KChIP2 alleles. For this purpose we performed whole-cell patch-clamp experiments on isolated left ventricular cardiomyocytes from male and female mice which had different KChIP2 genotypes; i.e., wild-type (KChIP2^+/+^), heterozygous knockout (KChIP2^+/-^) or complete knockout of KChIP2 (KChIP2^-/-^). We found in both sexes a KChIP2 gene dosage effect (i.e., a proportionality between number of alleles and phenotype) on I_to_ magnitude, however, concerning other I_to_ properties, KChIP2^+/-^ resembled KChIP2^+/+^. Only in the total absence of KChIP2 (KChIP2^-/-^) we observed a slowing of I_to_ kinetics, a slowing of recovery from inactivation and a negative shift of a portion of the voltage dependence of inactivation. In a minor fraction of KChIP2^-/-^ myocytes I_to_ was completely lost. The distinct KChIP2 genotype dependences of I_to_ magnitude and inactivation kinetics, respectively, seen in cardiomyocytes were reproduced with two-electrode voltage-clamp experiments on *Xenopus* oocytes expressing Kv4.2 and different amounts of KChIP2. Our results corroborate the critical role of KChIP2 in controlling I_to_ properties. They demonstrate that the Kv4.2/KChIP2 interaction in cardiomyocytes is highly dynamic, with a clear KChIP2 gene dosage effect on Kv4 channel surface expression but not on inactivation gating.

## Introduction

A transient outward current (I_to_) causes the initial (phase 1) repolarization of the action potential in ventricular cardiomyocytes [1], thereby controlling Ca^2+^ entry and excitation-contraction coupling [2]. A large portion of I_to_ is mediated by voltage-dependent K^+^ (Kv) channels belonging to the Kv4 subfamily [3–7] associated with cytoplasmic Kv Channel Interacting Proteins (KChIPs; [6, 8, 9]). Different genes are coding for Kv4.1, Kv4.2 and Kv4.3 channel subtypes, respectively, with two splice variants of Kv4.3 [10]. There are also different KChIP genes coding for KChIP1, KChIP2, KChIP3 and KChIP4, respectively, with different splice variants for each gene [11, 12]. The molecular correlates of I_to_ expression in cardiomyocytes have been extensively studied in several species (reviewed in [13]). Generally, there is abundant Kv4.2 and/or Kv4.3 expression and abundant KChIP2 expression, with different relative amounts depending on species, cardiac tissue and transmural cell layer [13].

Heterologous coexpression of Kv4 channels with KChIPs has been shown to modulate both channel expression and gating [8, 14, 15]. Binding of KChIPs increases Kv4 channel surface expression by various mechanisms, including promoted tetrameric assembly, a chaperone-like effect on trafficking, increased protein stability and promoted surface retention, all leading to a tremendous increase in measured current amplitudes [8, 14, 16–19]. Furthermore, KChIP binding modulates Kv4 channel inactivation properties, resulting in a slowing of macroscopic inactivation, an acceleration of recovery from inactivation and a positive shift in the voltage dependence of inactivation [8, 14, 15].

Two findings have initially led to the conclusion that KChIP2 plays an essential role in I_to_ expression in cardiomyocytes: The first finding has to do with the transmural I_to_ gradient observed in the ventricular free wall in many species, with larger I_to_ amplitudes usually found in epicardial than in endocardial myocytes [13]. This gradient has been reported to correlate with KChIP2 rather than Kv4 mRNA expression in humans and dogs [20]. The second finding concerns the I_to_ amplitudes measured in ventricular myocytes isolated from KChIP2 knockout mice [9]. I_to_ has been reported to be completely absent in myocytes of homozygous (KChIP2^-/-^) and reduced by approximately half in myocytes of heterozygous (KChIP2^+/-^) knockout mice. The latter finding was considered a possible KChIP2 gene dosage effect on I_to_ expression [9]; i.e., there seemed to be a proportionality between the number of functional KChIP2 alleles and the phenotypic I_to_ expression. However, the putative KChIP2 gene dosage effect has not been studied further and its applicability to the kinetics and voltage dependence of inactivation as well as its gender dependence have not been examined. The present study was conducted to characterize the KChIP2 genotype dependence of I_to_ properties in more detail. Using the same KChIP2 knockout mouse line we set out to answer the following questions related to the KChIP2 dependence of I_to_: First, do all I_to_ properties show a KChIP2 gene dosage effect? Second, is there an absolute requirement of KChIP2 for the expression of I_to_? And third, are there gender-dependent differences regarding the role of KChIP2 in the control of I_to_ properties? Our results confirm that KChIP2 is a key regulator of I_to_ in both sexes. However, I_to_ is not completely lost in the majority of homozygous KChIP2 knockout myocytes but kinetically modified. Our results reveal a partial KChIP2 gene dosage effect on I_to_, because it is restricted to current magnitude.

## Materials and methods

### Ethical approval of animal procedures

This study was carried out in strict accordance with the local institutional guidelines after approval from local authorities (Institutional Animal Care and Use Committee: Behörde für Gesundheit und Verbraucherschutz, Hamburg, Germany; permit numbers: ORG349 and G119/15). Mice were sacrificed under isoflurane anesthesia, and frog surgery was performed under 3-aminobenzoate methanesulfonate anesthesia, and all efforts were made to minimize suffering.

### Mouse line and cardiomyocyte preparation

Wild-type and genetically modified C57BL/6 mice (10–12 weeks old) were used for cardiomyocyte preparation. Genetically modified mice carried a heterozygous (+/-) or a homozygous deletion (-/-) of the KChIP2 gene. The generation and reactivation of the KChIP2 knockout mouse line have been previously described [9, 21]. For our experiments the animals were obtained from the local animal facility, where they were held at room temperature (20–22°C), with food and water available *ad libitum*, and where they showed normal breeding. Genotyping was done based on tail biopsy. Mice were sacrificed by cervical dislocation under isoflurane anesthesia. The heart was excised, mounted on a temperature-controlled (37°C) Langendorff perfusion system and rinsed with a Ca^2+^-free solution (113 mM NaCl, 4.7 mM KCl, 0.6 mM KH_2_PO_4_, 0.6 mM Na_2_HPO_4_, 1.2 mM MgSO_4_, 12 mM NaHCO_3_, 10 mM KHCO_3_, 30 mM taurine, 5.5 mM glucose, 10 mM HEPES, pH 7.46). Then the heart was digested for 9–10 min with the same solution containing 0.1 mg/ml Liberase Blendzyme (Roche). Epicardial tissue was obtained from the left ventricular free wall and dissociated into single cells. Ca^2+^ was stepwise reintroduced to a final concentration of 0.5–1 mM.

### Patch-clamp recordings

Whole-cell patch-clamp recordings were performed on individual myocytes at room temperature (20–22°C). The myocytes were bathed in extracellular solution containing 138 mM NaCl, 4 mM KCl, 1 mM CaCl_2_, 2 mM MgCl_2_, 0.33 NaH_2_PO_4_, 10 mM HEPES and 10 mM glucose (pH 7.3, NaOH). Patch pipettes were filled with internal solution containing 120 mM L-glutamate, 10 mM KCl, 2 mM K_2_-ATP, 2 mM MgCl_2_, 10 mM EGTA and 10 mM HEPES (pH 7.2, KOH). Recordings were done with an EPC-9 patch-clamp amplifier controlled by Pulse software (HEKA,). Pipette-to-bath resistance was between 2 and 2.5 MΩ, and the liquid junction potential (13 mV) was corrected online. Myocytes were held at -80 mV and outward currents were activated by depolarizing test pulses of different length to +40 mV. Series resistance (usually between 3 and 5 MΩ) was maximally compensated (70–80%). In order to inactivate a fraction of the currents conditioning prepulses were applied before the test pulse. Recovery from inactivation was studied with a double-pulse protocol (long control pulse and brief test pulse separated by an interpulse interval of different length at -80 mV). The voltage dependence of inactivation was studied with a variable prepulse protocol (long conditioning prepulse to different voltages between -100 and 0 mV followed by a brief test pulse).

### *Xenopus* oocyte preparation and RNA injection

Kv4.2 channels were coexpressed in *Xenopus* oocytes with different amounts of KChIP2. *Xenopus* frogs were obtained from Nasco (Fort Atkinson, WI). The oocytes were surgically obtained under temporary ethyl 3-aminobenzoate methanesulfonate anesthesia. For this purpose 1.2 g of the anesthetic were disolved in 1 liter tap water together with 25 ml of a 0.5 M Na_2_HPO_4_ solution to keep the pH in a neutral range [22]. After surgical removal the oocytes were treated with collagenase for 3–4 h. After 12 h or later 50 nl of an aqueous cRNA solution were injected into individual oocytes with a Nanoliter 2000 microinjector (World Precision Instruments). The amount of Kv4.2 cRNA was always 1 ng per oocyte, whereas the amount of KChIP2 cRNA was varied between 0 and 12.8 ng per oocyte.

### Two-electrode voltage-clamp recordings

Two-electrode voltage-clamp recordings from individual oocytes expressing Kv4.2/KChIP2 channels were done at room temperature (20–22°C) between 24 h and 96 h after cRNA injection. Voltage-clamp was achieved with a TurboTec-03 amplifier (npi) controlled by Pulse software (HEKA). Microelectrodes were filled with 3 M KCl and the oocytes were bathed in a solution containing 91 mM NaCl, 2 mM KCl, 1.8 mM CaCl_2_, 1 mM MgCl_2_ and 5 mM HEPES (pH 7.4, NaOH). Oocytes were held at -80 mV, and currents were activated by voltage pulses to +40 mV. The recovery from inactivation at -80 mV was studied with a double-pulse protocol.

### Data analysis

**Fig 1:**
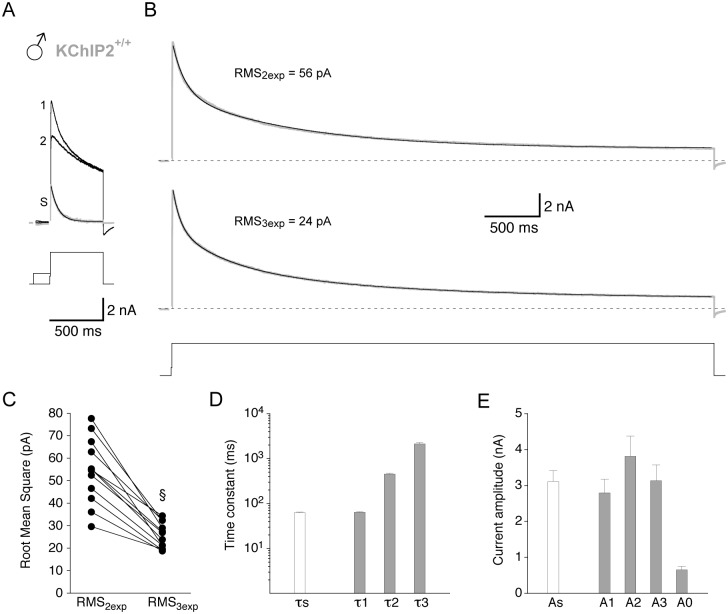
Isolation of I_to_ amplitude and inactivation kinetics. Whole-cell patch-clamp recordings were taken from individual murine cardiomyocytes isolated from the left ventricular free wall. Outward currents were activated by depolarizing voltage pulses from -80 to +40 mV. Inward sodium currents (not shown) were inactivated by a brief (8 ms) prepulse to -50 mV. Voltage protocols are shown below current traces. **A.** Prepulse-inactivation-subtraction method to isolate I_to_ in a male wild-type (KChIP2^+/+^) myocyte. A fraction of current was optionally inactivated by a 160 ms prepulse to -40 mV. Subtraction of the remaining outward current (2) from the compound outward current (obtained without the 160 ms prepulse, 1) yielded a rapidly inactivating current trace (S), referred to as I_to_. The inactivation of the I_to_ trace was fitted by a single-exponential function. **B.** Compound outward current recorded from the same myocyte as in A during a 5 s voltage pulse to +40 mV (dotted lines represent zero current). For the same current trace the decay was fitted by a double-exponential (2exp, top) and a triple-exponential function (3exp, bottom). The root mean square (RMS) values of the fits are indicated. **C.** Corresponding RMS_2exp_ and RMS_3exp_ values obtained for 12 male KChIP2^+/+^ myocytes. RMS_3exp_ values were significantly smaller than RMS_2exp_ values (§, Student's paired *t*-test); i.e. the triple-exponential fit was more accurate. **D.** Mean time constants obtained by fitting the decay of isolated I_to_ traces with a single-exponential function (τs, empty bar) and by fitting the compound outward current decay with a triple-exponential function (τ1, τ2 and τ3, grey bars). Note the close match between τs and τ1. **E.** Mean peak amplitude of the isolated I_to_ traces (empty bar) and amplitudes of the individual time constants obtained by triple-exponential fitting (A1, A2 and A3, grey bars). The amplitude of the non-inactivating current component (A0, right grey bar) was obtained by fitting P/n leak-subtracted current traces (not shown).

Acquired current traces were analysed with Pulsefit (HEKA), and further data analysis and statistical tests were done with Kaleidagraph (Synergy Software). Current traces with a monophasic rapid decay, assigned to I_to_, were generated with the prepulse-inactivation-subtraction method applied to cardiomyocytes ([23]; prepulse voltage -40 mV, prepulse duration 160 ms; see Fig 1A and S1 Fig). The decay of these current traces was fitted with a single-exponential function. The decay of compound outward currents obtained with 5 s long depolarizing pulses was fitted with a double- or a triple-exponential function. Root mean square (RMS) values in pA were used as a measure of fit accuracy. For each time constant (τx) an absolute amplitude (Ax) was provided by the fit. In our myocyte recordings τ1 and A1 obtained by triple-exponential fitting were assigned to I_to_. We calculated relative amplitudes (relAx) in %, and, based on the whole-cell capacitance (Cap), current densities (Dx) in pA/pF (Dx = Ax / Cap). Based on $\sum_{X=2}^{3}Ax$(double-expoential fit) or $\sum_{X=1}^{3}Ax$ (triple-exponential fit), plus a leak corrected non-inactivating current component A0 (P/n method), we also calculated the total amplitude (AΣ) and density (DΣ) of the compound outward current. Both recovery from inactivation and the voltage dependence of inactivation were analyzed for the compound outward current neglecting A0 and leak current. Recovery kinetics were analyzed by fitting the data with a single- or a double-exponential function. Inactivation curves were obtained by fitting the data with a single or the sum of two Boltzmann-functions. Data are presented as mean ± SEM. Statistical significance between two groups was calculated by Student’s *t*-test. For multiple comparisons, one-way analysis of variance (ANOVA) with Tukey's post-hoc test was performed. Differences with p < 0.05 were considered statistically significant. The concomitance of significant differences between KChIP2^+/+^ and KChIP2^+/-^, and between KChIP2^+/-^ and KChIP2^-/-^ (subject to a proportionality between the number of functional KChIP2 alleles and the phenotypic value) is referred to as a KChIP2 gene dosage effect on a given parameter.

## Results

### Isolation of I_to_

The present study was performed to characterize in detail a possible KChIP2 gene dosage effect on I_to_ properties and its gender dependence. I_to_ properties included current magnitude (i.e., amplitude and density), decay and recovery kinetics and the voltage dependence of inactivation. We studied individual myocytes isolated from epicardial tissue of the left ventricular free wall from male and female mice with different KChIP2 genotypes; i.e., KChIP2^+/+^, KChIP2^+/-^ and KChIP2^-/-^. For the detection of KChIP2 genotype- and/or gender-dependent differences a method both reliable and sensitive was needed for the isolation of I_to_. Initially we applied both the prepulse-inactivation-subtraction method [23] and a method based on the analysis of the initial decay component derived from multi-exponential fitting of the compound outward current ([24–26]; see Materials and Methods). Fig 1 demonstrates the complementary application of the two methods to male KChIP2^+/+^ myocytes. The prepulse-inactivation-subtraction method (Fig 1A, see also S1 Fig) yielded rapidly inactivating current traces, assigned to I_to_ at +40 mV, with a mean peak amplitude (As) of 3.11 ± 0.30 nA (n = 12; Fig 1E). Single-exponential fitting of these traces yielded a mean time constant of inactivation (τs) of 62.2 ± 2.1 ms (n = 12; Fig 1D). We also applied 5 s voltage pulses to +40 mV and fitted the decay of the compound outward currents with a double- and a triple-exponential function (Fig 1B). The triple-exponential function yielded better fits than the double-exponential function (RMS_2exp_ = 54.4 ± 4.2 pA, RMS_3exp_ = 25.5 ± 1.6 pA, n = 12, p<0.0001, Student's paired *t*-test; Fig 1B and 1C). The time constants obtained with triple-exponential fitting were τ1 = 63.5 ± 2.4 ms, τ2 = 449 ± 19 ms and τ3 = 2107 ± 158 ms (n = 12). Notably, the time constant of the fastest decay component (τ1) was virtually identical to the one obtained with the prepulse-inactivation-subtraction method (τs; Fig 1D). Based on this finding and the higher fit accuracy we decided to use triple-exponential fitting, whenever appropriate, for our further analyses of compound outward current decay kinetics in cardiomyocytes. Accordingly, the time constant τ1 and its amplitude A1 from the triple-exponential fits were assigned to I_to_. Similar to τ1 the I_to_ amplitude determined with triple-exponential fitting (A1 = 2.79 ± 0.38, n = 12) was very similar to the one obtained with the prepulse-inactivation-subtraction method (As; Fig 1E; no statistical test performed). The fit results (mean time constants and amplitudes) for male myocytes are summarized in Table 1, together with mean RMS values (in pA) for the double- and triple-exponential fits, relative amplitudes of the individual time constants (relAx) in %, whole-cell capacitance (Cap) in pF and current densities (Dx) in pA/pF, as well as the total amplitude and density (AΣ and DΣ, respectively) of the compound outward current at +40 mV. Taken together, comparable to the prepulse-inactivation-subtraction results (see S1 Table), triple-exponential fitting yielded an I_to_ amplitude of ~3 nA with an inactivation time constant of ~60 ms in male KChIP2^+/+^ myocytes. The absolute amplitudes and densities of the fast (I_to_-related), the intermediate and the slow component, as well as their relative contributions to the compound outward current decay (30%, 38% and 32%, respectively) were more or less balanced. The non-inactivating current component A0 was smaller than A1, A2 and A3 (see Fig 1E and Table 1).

**Table 1:** Data summary for male cardiomyocytes.

♂	KChIP2^+/+^	KChIP2^+/-^	KChIP2^-/-^	
	3exp	3exp	3exp	*2exp*
RMS_2exp_ (pA)	54.4 ± 4.2	44.4 ± 2.2	25.8 ± 1.3	*25*.*9 ± 3*.*1*
RMS_3exp_ (pA)	25.5 ± 1.6 ^§^	22.0 ± 0.8 ^§^	20.0 ± 0.9 ^§^	*21*.*2 ± 1*.*5*
**Current kinetics and magnitudes**				
τ1 (ms)	63.5 ± 2.4	61.7 ± 3.0	81.7 ± 4.9 **	-
τ2 (ms)	449 ± 19	436 ± 15	451 ± 26	*444 ± 29*
τ3 (ms)	2107 ± 158	2218 ± 115	2274 ± 177	*2154 ± 274*
A1 (nA)	2.79 ± 0.38	1.77 ± 0.12 *	0.75 ± 0.11 **	-
A2 (nA)	3.81 ± 0.56	3.00 ± 0.25	2.28 ± 0.27 *	*3*.*32 ± 0*.*53*
A3 (nA)	3.13 ± 0.44	2.42 ± 0.18	2.13 ± 0.21	*2*.*72 ± 0*.*32*
A0 (nA)	0.65 ± 0.10	0.25 ± 0.05 *	0.30 ± 0.06 *	*0*.*55 ± 0*.*11*
AΣ (nA)	10.4 ± 0.79	7.43 ± 0.43 *	5.46 ± 0.48 **	*6*.*59 ± 0*.*87*
relA1 (%)	30 ± 5	26 ± 2	16 ± 2 **	-
relA2 (%)	38 ± 4	40 ± 2	42 ± 2	*54 ± 3*
relA3 (%)	32 ± 3	34 ± 1	42 ± 2	*46 ± 3*
Cap (pF)	189 ± 12	169 ± 8	170 ± 13	*167 ± 6*
D1 (pA/pF)	15.6 ± 2.4	11.1 ± 0.8 *	4.9 ± 0.7 **	-
D2 (pA/pF)	20.3 ± 2.7	18.3 ± 1.5	14.2 ± 1.7	*19*.*7 ± 2*.*8*
D3 (pA/pF)	16.4 ± 1.9	14.4 ± 0.9	12.9 ± 0.9	*16*.*3 ± 1*.*7*
D0 (pA/pF)	3.4 ± 0.5	1.4 ± 0.3 *	2.0 ± 0.4	*3*.*2 ± 0*.*5*
DΣ (pA/pF)	55.6 ± 3.6	45.2 ± 2.3	34.0 ± 2.9 **	*39*.*2 ± 4*.*6*
	(n = 12)	(n = 38)	(n = 19)	*(n = 7)*
**Recovery from inactivation**				
τ_rec_1 (ms)	94.5 ± 5.4	88.6 ± 5.7	381 ± 43 **	*245 / 730*
τ_rec_2 (ms)	1490 ± 145	1210 ± 69	2296 ± 215 **	*1825 / 3736*
A_rec_1 (%)	63 ± 2	56 ± 6	58 ± 2	*59 / 65*
A_rec_2 (%)	37 ± 2	44 ± 2	42 ± 3	*41 / 35*
	(n = 9)	(n = 36)	(n = 11)	*(n = 2)*
**Voltage dependence of inactivation**				
V_1/2_1 (mV)	-52.8 ± 1.4	-54.1 ± 0.8	-69.7 ± 5.4 **	-
V_1/2_2 (mV)	-36.1 ± 3.5	-34.1 ± 0.9	-34.9 ± 1.4	*-33*.*0 ± 2*.*8*
A_V_1 (%)	63 ± 8	52 ± 4	10 ± 1 **	-
A_V_2 (%)	37 ± 8	48 ± 4	90 ± 1 **	-
	(n = 11)	(n = 31)	(n = 11)	*(n = 3)*

Analysis results obtained for current kinetics and magnitudes, recovery from inactivation and voltage dependence of inactivation for male myocytes with different KChIP2 genotypes.

* significantly different from KChIP2^+/+^;

** significantly different from both KChIP2^+/+^ and KChIP2^+/-^ (one way ANOVA);

^§^ significantly different from RMS_2exp_ (paired Student's *t*-test); abbreviations are explained in the text.

### KChIP2 genotype dependence of I_to_ properties in male myocytes

**Fig 2:**
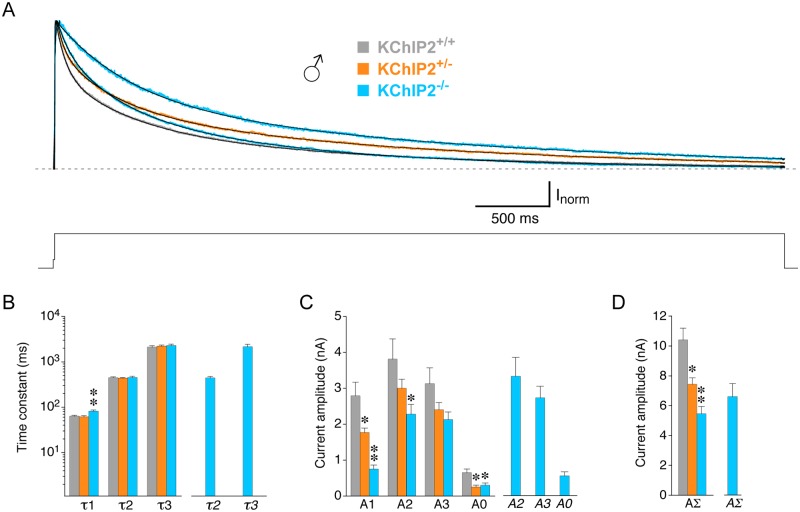
Multi-exponential fitting of compound outward current decay in male myocytes with different KChIP2 genotypes. Outward currents were activated by 5 s voltage pulses from -80 to +40 mV in male KChIP2^+/+^, KChIP2^+/-^ and KChIP2^-/-^ myocytes. The voltage protocol is shown below the current traces. **A.** Representative current traces for the different KChIP2 genotypes were normalized to peak and only the inactivating current components are shown (dotted line represents non-inactivating current level). Current decay kinetics in KChIP2^+/+^ (grey), KChIP2^+/-^ (orange) and most KChIP2^-/-^ myocytes (19 out of 26, blue, fast decay) were best described by a triple-exponential function. In some KChIP2^-/-^ myocytes (7 out of 26, blue, slow decay) a double-exponential function was sufficient. **B.** Mean time constants (τ1, τ2 and τ3) obtained with a triple-exponential function for male KChIP2^+/+^ (grey bars), KChIP2^+/-^ (orange bars) and most KChIP2^-/-^ myocytes (19 out of 26, blue bars), and mean time constants obtained with a double-exponential function for 7 out of 26 male KChIP2^-/-^ myocytes (*τ2* and *τ3*, separate blue bars on the right). **C.** Mean amplitudes of the individual time constants obtained by triple-exponential (A1, A2 and A3) and double-exponential fitting (*A2* and *A3*, separate bars on the right), and mean amplitudes of the corresponding non-inactivating current components (A0, *A0*). Note the KChIP2 gene dosage effect on A1. **D.** Mean total amplitudes of the compound outward current (AΣ, *AΣ*). The KChIP2 gene dosage effect observed for A1 is also reflected in AΣ (* significantly different from KChIP2^+/+^; ** significantly different from both KChIP2^+/+^ and KChIP2^+/-^; one-way ANOVA).

Fig 2A shows the inactivating components of normalized current traces obtained with a 5 s voltage pulse to +40 mV from male myocytes which had different KChIP2 genotypes. Currents from wild-type (KChIP2^+/+^) myocytes showed the fastest decay kinetics, whereas currents from heterozygous (KChIP2^+/-^) and homozygous KChIP2 knockout myocytes (KChIP2^-/-^) decayed more slowly. Notably, in some KChIP2^-/-^ myoctes (7 out of 26) current decay was extremely slow. Similar to KChIP2^+/+^, outward current decay kinetics were best described by a triple-exponential function in all KChIP2^+/-^ and most KChIP2^-/-^ myocytes (19 out of 26). The fit results are shown in Fig 2B and 2C (see also Table 1). In KChIP2^+/-^ myocytes the I_to_ kinetics (τ1 = 61.7 ± 3.0 ms, n = 38) were not significantly different from KChIP2^+/+^, whereas KChIP2^-/-^ I_to_ kinetics (τ1 = 81.7 ± 4.9 ms, n = 19) were slower. I_to_ amplitude, on the other hand, was smaller, compared to KChIP2^+/+^, in KChIP2^+/-^ myocytes (A1 = 1.77 ± 0.12 nA, n = 38) and even smaller in KChIP2^-/-^ myocytes (A1 = 0.75 ± 0.11 nA, n = 19). Neither the intermediate nor the slow decay kinetics showed a dependence on the KChIP2 genotype (Fig 2B), however, A2 was reduced in a KChIP2 genotype-dependent manner, as was the amplitude of the non-inactivating current component A0 (Fig 2C). Accordingly, the total amplitude of the compound outward current (AΣ) was also reduced in a KChIP2 genotype-dependent manner (Fig 2D and Table 1). In the KChIP2^-/-^ myocytes with an extremely slow current decay, the kinetics were well described by a double-exponential function (Fig 2A and Table 1). In these KChIP2^-/-^ myocytes the time constants of the fast and slow decay component obtained with double-exponential fitting were virtually identical to the time constants of the intermediate and slow component obtained for all other myocytes with triple-exponential fitting (Fig 2B). However, their amplitudes appeared larger than the corresponding values obtained in the other KChIP2^-/-^ myocytes (Fig 2C), and therefore, the total amplitude AΣ was comparable in all male KChIP2^-/-^ myocytes (Fig 2D and Table 1; no statistical tests performed). Taken together, in male myocytes I_to_ amplitude showed a clear KChIP2 gene dosage effect, with a reduction from ~ 3 nA in KChIP2^+/+^ to ~ 2 nA in KChIP2^+/-^ and to ~ 1 nA in KChIP2^-/-^. I_to_ density was equally affected since whole-cell capacitance (i.e., cell size) was not significantly different among KChIP2 genotypes (Table 1). In male KChIP2^-/-^ myocytes I_to_ had an increased inactivation time constant (~80 ms), and I_to_ only accounted for 16% of the compound outward current decay, while either of the two remaining components accounted for 42% (Table 1). In a fraction of male KChIP2^-/-^ myocytes I_to_ was apparently lost.

**Fig 3:**
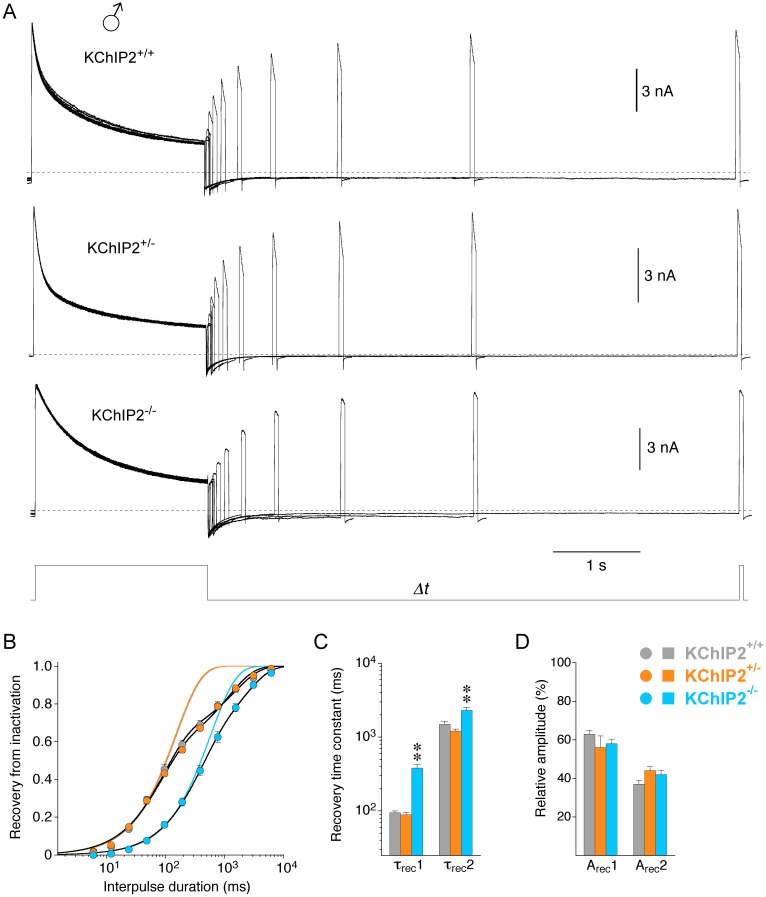
Recovery from inactivation in male myocytes with different KChIP2 genotypes. Recovery from inactivation at -80 mV was studied in male myocytes using a double-pulse protocol with interpulse durations between 6 ms and 6.14 s (*Δt*) followed by a brief test pulse (voltage protocol shown below current traces). **A.** Representative current families, recorded with the double-pulse protocol for the different KChIP2 genotypes (dotted lines represent zero current). **B.** Relative peak current amplitudes obtained with the brief test pulse were plotted against interpulse duration for the different KChIP2 genotypes (KChIP2^+/+^: grey, KChIP2^+/-^: orange, KChIP2^-/-^: blue), and the data were fitted with a double-exponential function. Recovery kinetics for KChIP2^+/+^ and KChIP2^+/-^ were virtually identical. Lines without symbols represent single-exponential functions fitted to a fraction of the data points and forced to reach 1 (orange and grey line superimpose). They represent the fast recovery component and emphasize the necessity of a double-exponential function to adequately fit the recovery kinetics for all KChIP2 genotypes. **C.** Mean recovery time constants (τ_rec_1 and τ_rec_2) obtained by double-exponential fitting (** significantly different from both KChIP2^+/+^ and KChIP2^+/-^; one-way ANOVA). **D.** Mean relative amplitudes of the recovery time constants (A_rec_1 and A_rec_2).

Next we studied the recovery of the compound outward current from inactivation in male myocytes with a double-pulse protocol (Fig 3A; see Materials and Methods). For all three KChIP2 genotypes the recovery kinetics were best described by a double-exponential function (Fig 3B). In male myocytes KChIP2^+/+^ and KChIP2^+/-^ recovery kinetics were virtually identical (KChIP2^+/+^: τ_rec_1 = 94.5 ± 5.4 ms, 63%; τ_rec_2 = 1490 ± 145 ms, 37%; n = 9; KChIP2^+/-^: τ_rec_1 = 88.6 ± 5.7 ms, 56%; τ_rec_2 = 1210 ± 69 ms, 44%; n = 36; Fig 3B–3D). KChIP2^-/-^ myocytes showed slower recovery kinetics due to larger recovery time constants with more or less unchanged relative contributions (τ_rec_1 = 381 ± 43 ms, 58%; τ_rec_2 = 2296 ± 215 ms, 42%; n = 11; Fig 3B and 3D and Table 1). The data obtained from two KChIP2^-/-^ myocytes with an apparent loss of I_to_ suggested similar recovery kinetics in all male KChIP2^-/-^ myocytes (Table 1).

**Fig 4:**
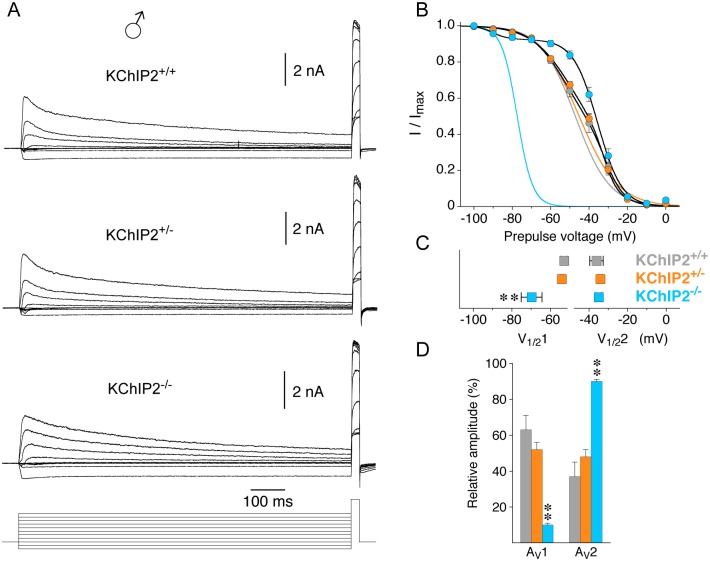
Voltage dependence of inactivation in male myocytes with different KChIP2 genotypes. The voltage dependence of inactivation was studied in male myocytes with a variable prepulse protocol. The prepulse (1 s, between -100 and 0 mV) was followed by a brief test pulse (voltage protocol shown below current traces). **A.** Representative current families, recorded with the variable prepulse protocol, for the different KChIP2 genotypes. **B.** Normalized peak current amplitudes obtained with the test pulse (I / I_max_) were plotted against prepulse voltage for the different KChIP2 genotypes (KChIP2^+/+^: grey, KChIP2^+/-^: orange, KChIP2^-/-^: blue), and the data were fitted with the sum of two Boltzmann-functions. The voltage dependences for KChIP2^+/+^ and KChIP2^+/-^ were virtually identical. Lines without symbols represent single Boltzmann-functions fitted to a fraction of the data points and forced to reach 0. They extrapolate the more negative portion of the voltage dependences and emphasize the necessity of the sum of two Boltzmann-functions to adequately fit the voltage dependence of inactivation. **C.** Mean values of the voltages of halfmaximal inactivation obtained for the two components (V_1/2_1 and V_1/2_2) obtained with the sum of two Boltzmann-functions. Note that V_1/2_1 was shifted to more negative potentials for KChIP2^-/-^, whereas V_1/2_2 values were similar in all KChIP2 genotypes. **D.** Mean relative amplitudes of the two components defined by V_1/2_1 and V_1/2_2, respectively (A_v_1 and A_v_2); ** significantly different from both KChIP2^+/+^ and KChIP2^+/-^; one-way ANOVA).

The voltage dependence of compound outward current inactivation of male myocytes was studied with a variable prepulse protocol (Fig 4A; see Materials and Methods). For all three KChIP2 genotypes the data were best described by the sum two Boltzmann-functions (Fig 4B), defining a first (more negative) and a second (less negative) component. Similar to the recovery kinetics, the voltage dependence of inactivation in male myocytes was almost identical for KChIP2^+/+^ and KChIP2^+/-^ (KChIP2^+/+^: V_1/2_1 = -52.8 ± 1.4 mV, 63%; V_1/2_2 = -36.1 ± 3.5 mV, 37%; n = 11; KChIP2^+/-^: V_1/2_1 = -54.1 ± 0.8 mV, 52%; V_1/2_2 = -34.1 ± 0.9 mV, 48%; n = 31; Fig 4B–4D). Intriguingly, male KChIP2^-/-^ myocytes showed a negative shift in V_1/2_1 (-69.7 ± 5.4 mV) with no change in V_1/2_2 (-34.9 ± 1.4 mV, n = 11; Fig 4C), and the component defined by V_1/2_1 only accounted for 10% (Fig 4D and Table 1). In the male KChIP2^-/-^ myocytes with an apparent loss of I_to_ a single Boltzmann-function was sufficient to describe the voltage dependence of inactivation and the resultant V_1/2_ value (-34.9 ± 1.4 mV, n = 3) resembled V_1/2_2 obtained for all other myocytes with the sum of two Boltzmann-functions (Table 1).

### I_to_ properties in female myocytes

**Fig 5:**
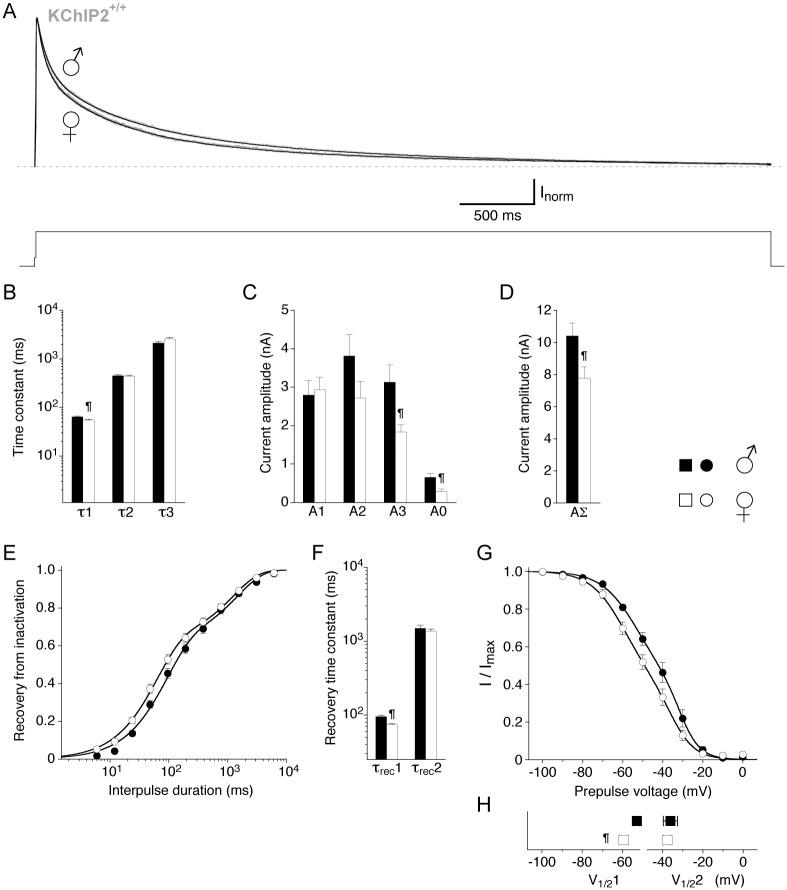
Comparison of outward current inactivation properties in male and female wild-type myocytes. For the compound outward current in male and female wild-type (KChIP2^+/+^) myocytes the kinetics of macroscopic inactivation, the kinetics of recovery from inactivation and the voltage dependence of inactivation were compared. Data from male myocytes are depicted as black symbols, data from female myocytes as empty symbols. **A.** Representative outward current traces from a male and a female KChIP2^+/+^ myocyte, respectively, recorded during a 5 s voltage pulse to +40 mV (voltage protocol shown below current traces; dotted line represents non-inactivating current level). For both sexes KChIP2^+/+^ outward current decay kinetics were best described by a triple-exponential function. Note the slightly faster decay kinetics in the female myocyte. **B.** Mean time constants obtained with a triple-exponential function (τ1, τ2 and τ3). **C.** Mean amplitudes of the individual time constants obtained by triple-exponential fitting (A1, A2 and A3) and the amplitude of the non-inactivating current component (A0). **D.** Mean total amplitude of the compound outward current (AΣ). **E.** Recovery data fitted by a double-exponential function. Note that for female KChIP2^+/+^ myocytes the recovery from inactivation was apparently faster. **F.** Mean recovery time constants (τ_rec_1 and τ_rec_2) obtained by double-exponential fitting. **G.** Data describing the voltage dependence of inactivation fitted with the sum of two Boltzmann-functions. Note the apparent negative shift of the fitting curve in female relative to male KChIP2^+/+^ myocytes. **H.** Mean values for V_1/2_1 and V_1/2_2 obtained with the sum of two Boltzmann-functions (¶, unpaired Student’s *t*-test).

In the present study I_to_ properties were also examined for female myocytes (summarized in Table 2). Before we will describe their KChIP2 genotype dependence we compare the I_to_ properties found in male and female KChIP2^+/+^ myocytes. The data obtained from female myocytes displayed the same fit requirements. The triple-exponential fitting analysis method to isolate I_to_ from female myocytes (Fig 5A) yielded a time constant τ1 of 54.4 ± 2.3 ms (n = 16), smaller than the one found in male myocytes. The intermediate and slow decay kinetics showed no gender-dependent differences (Fig 5B and Table 2). In female myocytes the I_to_ amplitude A1 was 2.93 ± 0.33 nA (n = 16), comparable to male myocytes (Fig 5C), however, the amplitudes A3 and A0, and, accordingly, the total amplitude of the compound outward current AΣ were smaller than in male myocytes (Fig 5C and 5D and Table 2). Due to the smaller cell size of female myocytes, these differences are not reflected in the current densities (Table 2). The kinetics of recovery from inactivation appeared faster in female myocytes (Fig 5E). This was due to a smaller τ_rec_1 value (73.2 ± 3.3 ms, n = 13). The second recovery component (τ_rec_2 = 1365 ± 87 ms, n = 13) was not different from male myocytes (Fig 5F and Table 2). The voltage dependence of inactivation appeared negatively shifted in female myocytes (Fig 5G), which was due to a more negative V_1/2_1 value (-59.3 ± 2.1 mV, n = 11). The second (less negative) component of the voltage dependence (V_1/2_2 = -37.6 ± 1.9 mV, n = 11) was not different from male myocytes (Fig 5H and Table 2).

**Table 2:** Data summary for female cardiomyocytes.

♀	KChIP2^+/+^	KChIP2^+/-^	KChIP2^-/-^	
	3exp	3exp	3exp	*2exp*
RMS_2exp_ (pA)	50.4 ± 5.0	43.4 ± 2.0	36.5 ± 3.1	*18*.*8 ± 3*.*8*
RMS_3exp_ (pA)	20.3 ± 1.2 ^§^	20.3 ± 0.6 ^§^	20.8 ± 1.1 ^§^	*16*.*1 ± 2*.*1*
**Current kinetics and magnitudes**				
τ1 (ms)	54.4 ± 2.3 ^¶^	58.2 ± 1.3	70.3 ± 7.0 **	-
τ2 (ms)	436 ± 20	447 ± 10	442 ± 14	*241 ± 19*
τ3 (ms)	2544 ± 222	2654 ± 114	2399 ± 286	*2440 ± 367*
A1 (nA)	2.93 ± 0.33	1.94 ± 0.11 *	1.23 ± 0.15 **	-
A2 (nA)	2.72 ± 0.43	2.56 ± 0.19	2.87 ± 0.37	*0*.*74 ± 0*.*43*
A3 (nA)	1.83 ± 0.19 ^¶^	1.99 ± 0.09	1.96 ± 0.13	*1*.*99 ± 0*.*25*
A0 (nA)	0.28 ± 0.07 ^¶^	0.24 ± 0.03	0.38 ± 0.07	*0*.*16 ± 0*.*09*
AΣ (nA)	7.76 ± 0.72 ^¶^	6.74 ± 0.33	6.44 ± 0.61	*2*.*89 ± 0*.*67*
relA1 (%)	40 ± 3	31 ± 1 *	20 ± 2 **	-
relA2 (%)	35 ± 3	37 ± 1	45 ± 2 **	*24 ± 9*
relA3 (%)	25 ± 2	32 ± 1 *	35 ± 3 *	*76 ± 9*
Cap (pF)	142 ± 12 ^¶^	155 ± 5	172 ± 12	*196 ± 23*
D1 (pA/pF)	21.5 ± 2.2	13.1 ± 0.8 *	7.8 ± 1.1 **	-
D2 (pA/pF)	18.9 ± 2.4	16.9 ± 1.2	17.4 ± 2.1	*4*.*3 ± 2*.*9*
D3 (pA/pF)	12.9 ± 0.8	13.1 ± 0.5	11.9 ± 1.0	*10*.*5 ± 2*.*0*
D0 (pA/pF)	2.0 ± 0.5	1.5 ± 0.2	2.2 ± 0.3	*1*.*0 ± 0*.*6*
DΣ (pA/pF)	55.3 ± 3.1	44.7 ± 2.0 *	39.1 ± 3.9 *	*15*.*7 ± 5*.*3*
	(n = 16)	(n = 67)	(n = 14)	*(n = 3)*
**Recovery from inactivation**				
τ_rec_1(ms)	73.2 ± 3.3 ^¶^	71.0 ± 2.6	339 ± 29 **	*855*
τ_rec_2 (ms)	1365 ± 87	1156 ± 31	1792 ± 139 **	*2742*
A_rec_1 (%)	65 ± 2	53 ± 1 *	49 ± 4 *	*58*
A_rec_2 (%)	35 ± 2	47 ± 1 *	51 ± 4 *	*42*
	(n = 13)	(n = 61)	(n = 9)	*(n = 1)*
**Voltage dependence of inactivation**				
V_1/2_1 (mV)	-59.3 ± 2.1 ^¶^	-56.5 ± 1.4	-75.7 ± 4.3 **	*-90*.*9*
V_1/2_2 (mV)	-37.6 ± 1.9	-35.6 ± 0.6	-38.7 ± 1.2	*-40*.*8*
A_V_1 (%)	58 ± 6	52 ± 3	12 ± 2 **	*13*
A_V_2 (%)	42 ± 6	48 ± 3	88 ± 2 **	*87*
	(n = 11)	(n = 55)	(n = 9)	*(n = 1)*

Analysis results obtained for current kinetics and magnitudes, recovery from inactivation and voltage dependence of inactivation for female myocytes with different KChIP2 genotypes.

^¶^ significantly different from male myocytes (unpaired Student's *t*-test);

* significantly different from KChIP2^+/+^;

** significantly different from both KChIP2^+/+^ and KChIP2^+/-^ (one way ANOVA);

^§^ significantly different from RMS_2exp_ (paired Student's *t*-test); abbreviations are explained in the text.

**Fig 6:**
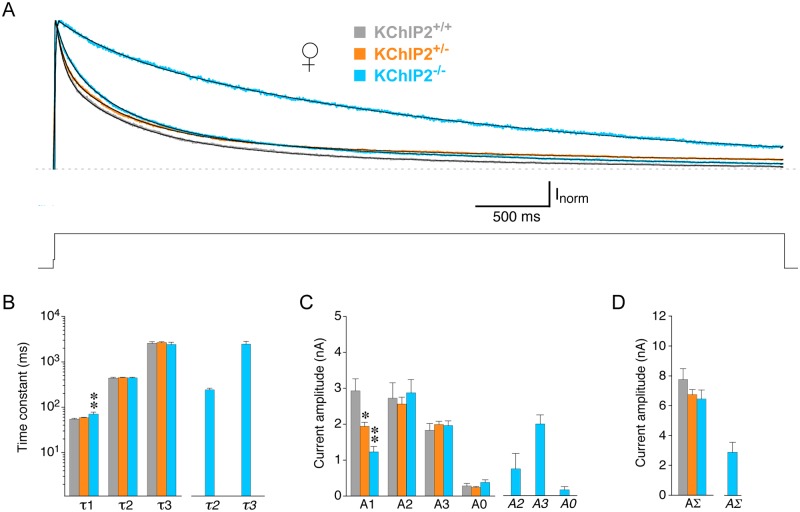
Analysis of macroscopic inactivation in female myocytes with different KChIP2 genotypes. Outward currents were activated by 5 s voltage pulses from -80 to +40 mV in female KChIP2^+/+^, KChIP2^+/-^ and KChIP2^-/-^ myocytes. **A.** Representative current traces for the different KChIP2 genotypes were normalized to peak and only the inactivating current components are shown (voltage protocol below current traces; dotted line represents non-inactivating current level). Current decay kinetics in KChIP2^+/+^ (grey), KChIP2^+/-^ (orange) and most KChIP2^-/-^ myocytes (14 out of 17, blue, fast decay) were best described by a triple-exponential function. In some KChIP2^-/-^ myocytes (3 out of 17, blue, slow decay) a double-exponential function was sufficient. **B.** Mean time constants obtained with a triple-exponential function (τ1, τ2 and τ3) for female KChIP2^+/+^ (grey bars), KChIP2^+/-^ (orange bars) and most KChIP2^-/-^ myocytes (14 out of 17, blue bars), and mean time constants obtained with a double-exponential function for 3 out of 17 female KChIP2^-/-^ myocytes (*τ2* and *τ3*, separate blue bars on the right). **C.** Mean amplitudes of the individual time constants obtained by triple-exponential (A1, A2 and A3) and double-exponential fitting (*A2* and *A3*, separate bars on the right) and mean amplitudes of the corresponding non-inactivating current components (A0, *A0*). Like in male myocytes, there was a gene dosage effect on A1 in female myocytes (* significantly different from KChIP2^+/+^; ** significantly different from both KChIP2^+/+^ and KChIP2^+/-^; one-way ANOVA). **D.** Mean total amplitudes of the compound outward current (AΣ, *AΣ*). The KChIP2 gene dosage effect observed for A1 is not reflected in AΣ in female myocytes.

Fig 6A shows the inactivating components of normalized current traces recorded at +40 mV from female myocytes with different KChIP2 genotypes. In general, the decay kinetics and KChIP2 genotype-specific features of outward currents in female myocytes were similar to the ones found for male myocytes: Currents from female KChIP2^+/+^ myocytes exhibited the fastest decay kinetics, while KChIP2^+/-^ and KChIP2^-/-^ showed slower decay kinetics. Outward current decay was best described by a triple-exponential function for all KChIP2 genotypes, but in a fraction of female KChIP2^-/-^ myocytes (3 out of 17) current decay was extremely slow and could be sufficiently well described by a double-exponential function (Fig 6A). The fit results for female myocytes are shown in Fig 6B and 6C. In female KChIP2^+/-^ myocytes the I_to_ kinetics (τ1 = 58.2 ± 1.3 ms, n = 67) were not significantly different from KChIP2^+/+^, whereas KChIP2^-/-^ I_to_ kinetics (τ1 = 70.3 ± 7.0 ms, n = 14) were slower (Fig 6B and Table 2). In female myocytes I_to_ amplitude also showed a clear KChIP2 gene dosage effect with A1 = 1.94 ± 0.11 nA (n = 67) for KChIP2^+/-^ and A1 = 1.23 ± 0.15 nA (n = 14) for KChIP2^-/-^ (Fig 6C and Table 2), which was also reflected in the current densities (Table 2). Also similar to male myocytes, neither the intermediate nor the slow decay kinetics showed a dependence on the KChIP2 genotype, however, in female myocytes this applied also to their amplitudes, including A0, and densities (Fig 6B and 6C and Table 2). Therefore, the total amplitude of the compound outward current AΣ was not significantly different among the three KChIP2 genotypes (Fig 6D and Table 2), albeit the corresponding densities proved to be significantly reduced in female KChIP2^+/-^ and KChIP2^-/-^ myocytes (Table 2). In the female KChIP2^-/-^ myocytes with an apparent loss of I_to_ the time constants of the fast and slow decay component obtained with double-exponential fitting also closely resembled the time constants of the intermediate and slow decay component obtained for all other myocytes with triple-exponential fitting (Fig 6B). However, the amplitude of the fast component obtained with double-exponential fitting was apparently reduced, and, accordingly, the total amplitude *AΣ* appeared smaller in these female KChIP2^-/-^ myocytes (Fig 6C and 6D; no statistical tests performed).

**Fig 7:**
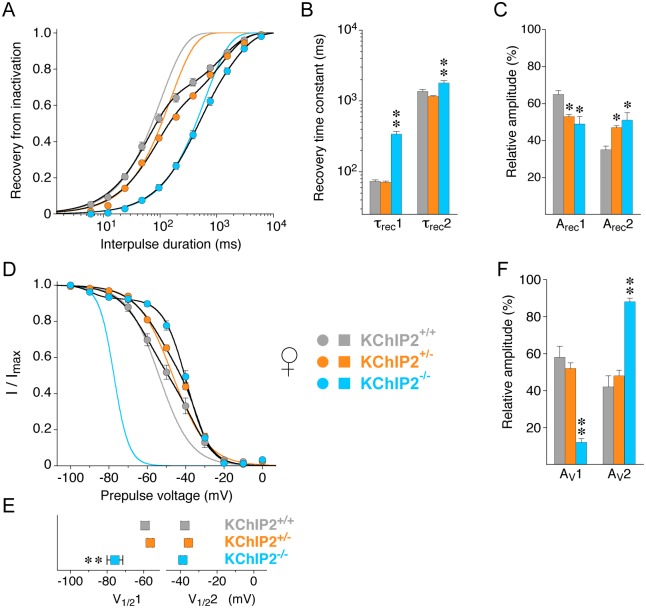
Recovery from inactivation and voltage dependence of inactivation in female myocytes with different KChIP2 genotypes. Recovery from inactivation and the voltage dependence of inactivation were studied in female KChIP2^+/+^ (grey symbols), KChIP2^+/-^ (orange symbols) and KChIP2^-/-^ myocytes (blue symbols). **A.** Recovery plots. Fitting curves represent double-exponential functions. Lines without symbols represent single-exponential functions fitted to a fraction of the data points (the fast component) and forced to reach 1. Note that in female myocytes a moderate apparent slowing of the recovery kinetics was observed for KChIP2^+/-^ compared to KChIP2^+/+^. **B.** Mean recovery time constants (τ_rec_1 and τ_rec_2) obtained by fitting the recovery kinetics with a double-exponential function. **C.** Mean relative amplitudes of recovery time constants for all KChIP2 genotypes (A_rec_1 and A_rec_2). **D.** Inactivation curves. The data were fitted with the sum of two Boltzmann-functions. Lines without symbols represent single Boltzmann-functions fitted to a fraction to the data points (the more negative portion) and forced to reach 0. **E.** Mean values for V_1/2_1 and V_1/2_2 obtained with the sum of two Boltzmann-functions. Note that V_1/2_2 values are similar in all KChIP2 genotypes. **F.** Mean relative amplitudes of the voltage dependences defined by V_1/2_1 and V_1/2_2 (A_v_1 and A_v_2); * significantly different from KChIP2^+/+^; ** significantly different from both KChIP2^+/+^ and KChIP2^+/-^; one-way ANOVA.

Like in male myocytes the kinetics of recovery of the compound outward current from inactivation in female myocytes were best described by the sum of two exponential functions for all three KChIP2 genotypes (Fig 7A). The recovery kinetics differed for female KChIP2^+/-^ and KChIP2^+/+^ myocytes. While the time constants in KChIP2^+/-^ (τ_rec_1 = 71.0 ± 2.6 ms, τ_rec_2 = 1156 ± 31 ms) were similar to KChIP2^+/+^ (Fig 7B), their relative contributions were shifted in favor of the slow recovery component (A_rec_1 = 53%, A_rec_2 = 47%, n = 61; Fig 7C). Female KChIP2^-/-^ and KChIP2^+/-^ myocytes shared this new distribution of relative amplitudes, however, in the KChIP2^-/-^ myocytes the recovery time constants were larger (τ_rec_1 = 339 ± 29 ms, 49%; τ_rec_2 = 1792 ± 139 ms, 51%, n = 9) resulting in a further slowing of the recovery kinetics (Fig 7A–7C and Table 2). Also similar to male myocytes, the voltage dependence of compound outward current inactivation was best described by the sum of two Boltzmann-functions for all KChIP2 genotypes in female myocytes (Fig 7D). No significant differences relative to KChIP2^+/+^ were detected for the KChIP2^+/-^ fit values (V_1/2_1 = -56.5 ± 1.4 mV, 52%; V_1/2_2 = -35.6 ± 0.6 mV, 48%; n = 55; Fig 7E and Table 2), although the corresponding inactivation curves apparently differ (Fig 7D). In KChIP2^-/-^ myocytes V_1/2_1 was shifted negative (-75.7 ± 4.3 mV) and the relative contribution was reduced to 12% in favor of the second (less negative) component (V_1/2_2 = -38.7 ± 1.2, 88%; n = 9; Fig 7E and 7F). Taken together, there were no qualitative differences between the data obtained for male and female KChIP2^+/+^ myocytes. However, I_to_ decay kinetics and the recovery from inactivation were faster and the first (more negative) component of the voltage dependence was more negative in female than in male KChIP2^+/+^ myocytes. Concerning the KChIP2 genotype dependence, the same effects, including a clear KChIP2 gene dosage effect on I_to_ magnitude but not on inactivation properties, were seen in male and female myocytes, albeit inactivation properties slightly differed for female KChIP2^+/+^ and KChIP2^+/-^ myocytes.

### Heterologous coexpression of Kv4.2 and KChIP2

**Fig 8:**
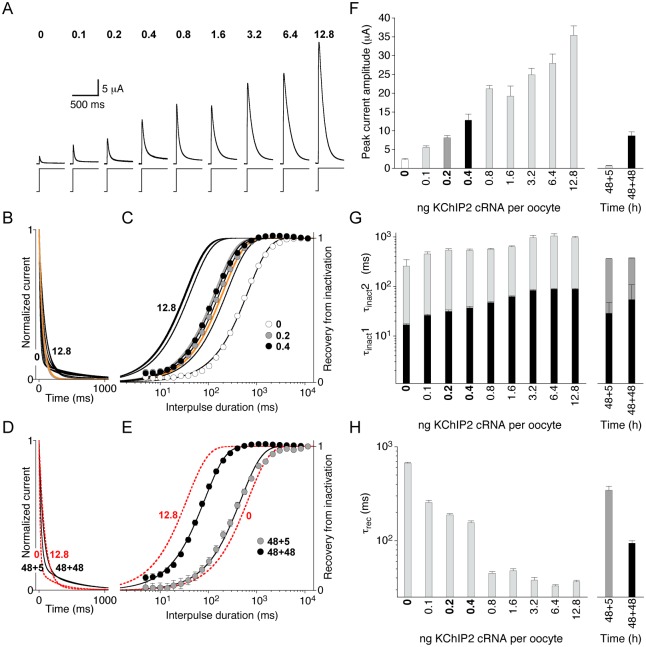
Expression of Kv4.2 and KChIP2 in *Xenopus* oocytes. Two-electrode voltage-clamp experiments were performed on individual oocytes following cRNA injection. **A.** Currents obtained with depolarizing voltage jumps from -80 to +40 mV (voltage protocols below traces) 48 h after cRNA injection. Different amounts of KChIP2 cRNA (ng per oocyte indicated above traces) were coinjected with a fixed amount of Kv4.2 cRNA (1 ng per oocyte). Note the tight correlation between KChIP2 cRNA amount and peak current amplitude. **B.** Idealized current traces based on the mean fit results obtained with a double-exponential function (Kv4.2/KChIP2-mediated currents with 0–12.8 ng KChIP2 cRNA coinjected: black) and based on the mean τ1 values from triple-exponential fits (i.e., the I_to_ component) of female myocytes (KChIP2^+/+^: grey, KChIP2^+/-^: orange). All currents were normalized to peak. **C.** Recovery from inactivation at -80 mV obtained for oocytes injected with different amounts of KChIP2 cRNA. Pooled recovery data are shown for oocytes injected with 1 ng Kv4.2 cRNA and 0 ng (empty symbols), 0.2 ng (grey symbols) and 0.4 ng KChIP2 cRNA (black symbols). Kv4.2/KChIP2 recovery kinetics were fitted by a single-exponential function. Black lines without symbols: Single-exponential fits for all other KChIP2 cRNA amounts (0.1, 0.8, 1.6, 3.2, 6.4 and 12.8 ng per oocyte); colored lines without symbols: Single-exponential functions representing the fast recovery component of female myocytes (see Fig 7A; KChIP2^+/+^: grey, KChIP2^+/-^: orange). **D.** Idealized current traces based on the same fitting procedures as the currents in B; black traces: currents obtained after consecutive injection of 1 ng Kv4.2 and 1 ng KChIP2 cRNA into the same oocyte; faster decay: 48 h Kv4.2 expression followed by 5 h KChIP2 expression (48+5); slower decay: 48 h Kv4.2 expression followed by 48 h KChIP2 expression (48+48). Red dotted traces represent the data for 0 and 12.8 ng KChIP2 cRNA from B. **E.** Recovery from inactivation for the two experimental paradigms (grey symbols: 48+5; black symbols: 48+48). The data were fitted by a single-exponential function. Red dotted lines: single exponential fits to the recovery data obtained with 0 and 12.8 ng KChIP2 cRNA per oocyte). Note the slow recovery component (~ 15%) in the 48+5 data, which was not captured by the single-exponential fit. **F.** Mean peak current amplitudes from A and from the experiments with consecutive cRNA injection (48+5 and 48+48). **G.** Inactivation time constants (τ_inact_1 and τ_inact_2) from B (0–12.8 ng KChIP2 cRNA per oocyte) and D (48+5 and 48+48). **H.** Recovery time constants (τ_rec_) from C (0–12.8 ng KChIP2 cRNA per oocyte) and E (48+5 and 48+48).

We asked whether the partial KChIP2 gene dosage effect on I_to_ seen in cardiomyocytes (i.e., heterozygous deletion of the KChIP2 gene has a strong effect on current magnitude but no or only a moderate effect on inactivation) can be reproduced with recombinant channel proteins in a heterologous expression system. For this purpose we expressed Kv4.2 in the absence or presence of KChIP2 in *Xenopus* oocytes, by injecting predefined cRNA amounts, and measured currents with the two-electrode voltage-clamp technique (see Materials and Methods). We were particularly interested in the dynamics of KChIP2 effects on Kv4.2-mediated current amplitudes and Kv4.2 inactivation kinetics. In order to establish appropriate experimental conditions, we first injected, into individual oocytes, 1 ng Kv4.2 cRNA either alone or in combination with 1 ng KChIP2 cRNA, measured the peak current amplitudes 24, 48, 72 and 96 h after cRNA injection and generated corresponding expression profiles (see S2A Fig). The results showed that using 1 ng per oocyte of Kv4.2 cRNA and measuring currents between 24 and 96 h after cRNA injection yields currents with decent amplitudes (between 1 and 40 μA) amenable to modulation by KChIP2 coexpression (5- to 7-fold gain in amplitude, see S2B Fig; [8, 14]). Next, we coinjected different amounts of KChIP2 cRNA (0–12.8 ng) with a fixed amount of 1 ng Kv4.2 cRNA into individual oocytes and measured currents 48 h after cRNA injection (Fig 8A). The peak amplitude of the currents showed a clear dependence on the amount of KChIP2 cRNA coinjected per oocyte (Fig 8F). The dependence was steep with lower amounts (e.g., 0.2 ng: 8.1 ± 0.6 μA, n = 9; 0.4 ng: 12.8 ± 1.6 μA, n = 7) and more shallow with higher amounts of KChIP2 cRNA (e.g., 3.2 ng: 24.9 ± 1.7 μA; 6.4 ng: 27.9 ± 2.5 μA, n = 9; see also S3A Fig). We also analysed macroscopic inactivation kinetics and the kinetics of recovery from inactivation (Fig 8B and 8C). For both parameters the KChIP2 dependence was obvious, with a slowing of macroscopic inactivation and an acceleration of recovery from inactivation in the presence of KChIP2, in accordance with published results [8, 14]. However, in contrast to the peak current amplitude (Fig 8F), macroscopic inactivation and recovery from inactivation showed only a vestigial dose dependence in the low to intermediate range of KChIP2 cRNA amounts and no dose dependence at all (i.e., the effects on inactivation gating were saturated) when cRNA amounts larger than 1.6 ng were coinjected with 1 ng Kv4.2 cRNA per oocyte (Fig 8B, 8C, 8G and 8H and S3 Fig). These findings are reminiscent of the KChIP2 gene dosage effect seen in cardiomyocytes for I_to_ magnitude but not for inactivation properties (see Discussion).

To further explore the dynamics of the Kv4.2/KChIP2 interaction we injected Kv4.2 and KChIP2 cRNAs (both 1 ng) at different time points into the same oocyte. After the initial injection of Kv4.2 cRNA, channels were allowed to assemble and get intalled in the plasma membrane in the absence of KChIP2 for 48 h. After this time KChIP2 cRNA was injected and currents were recorded either another 5 h (48+5) or another 48 h later (48+48). After 48 h of KChIP2 expression there was a clear gain in amplitude (compare Fig 8F and S2A Fig), macroscopic inactivation was slowed (Fig 8G), and recovery from inactivation was accelerated (Fig 8H). As expected, due to the KChIP2 cRNA amount of only 1 ng per oocyte, none of the KChIP2 effects on inactivation gating reached saturation. After 5 h of KChIP2 expression no gain in amplitude was apparent (Fig 8F), however, macroscopic inactivation was moderately slowed (Fig 8D and 8G) and recovery from inactivation moderately accelerated (Fig 8E and 8H). Notably, after 5 h the recovery kinetics exhibited a small (~ 15%) slow component, which was not captured by the single-exponential fit (Fig 8E), possibly representing a small amount of KChIP2-less Kv4.2 channels residing in the oocyte membrane. These results raise the question whether preexisting Kv4.2 channels in the oocyte membrane can be subsequently modified by KChIP2 (see Discussion).

## Discussion

We have shown in murine left ventricular myocytes a distinct KChIP2 gene dosage effect on I_to_ magnitude (i.e., amplitude and density). Inactivation properties (i.e., inactivation kinetics, kinetics of recovery from inactivation and voltage dependence of inactivation), on the other hand, were found to be similar in KChIP2^+/+^ and KChIP2^+/-^ myocytes. Only in KChIP2^-/-^ myocytes inactivation and recovery from inactivation were slowed and a portion of the voltage dependence of inactivation was shifted to more negative potentials. No qualitative differences concerning the KChIP2 genotype-dependent remodeling have been observed between male and female myocytes.

### Isolation of I_to_ properties: Technical aspects

In order to isolate I_to_ from the compound outward current in murine cardiomyocytes and to measure its amplitude and decay kinetics a prepulse-inactivation-subtraction method has been frequently used ([9, 23, 27, 28]; see also Fig 1A and S1 Fig of the present study). This method relies on the complete inactivation of I_to_-related potassium channels by a depolarizing prepulse which must not gate any other potassium channels. Therefore, both prepulse length and prepulse voltage are critical parameters. Typically, prepulse lengths between 75 ms [9] and 120 ms [27] and a prepulse voltage of -40 mV have been used. In the present study the prepulse-inactivation-subtraction method was applied to male myocytes using a prepulse length of 160 ms and a prepulse voltage of -40 mV (Fig 1A and S1 Fig). We obtained smaller I_to_ densities for wild-type myocytes (S1 Table) than reported in the literature based on the same method with test potentials between +30 and +50 mV (25–35 pA/pF; [9, 23, 27, 28]). Our isolated I_to_ kinetics (S1 Table) were comparable to the ones found by Tozakidou et al. (τ = 52 ms; [27]) but slower than the ones found by Brouillette et al. (τ = 28 ms; [23]). The prepulse-inactivation-subtraction method has several disadvantages: The requirements concerning prepulse length and prepulse voltage may depend on experimental conditions, such as the tissue from which the myocytes were isolated (e.g., apex vs. septum or endocard vs. epicard), age and gender of the mice, but also their genotype and genetic background (i.e., wild-type vs. mutant, different mouse strains). Therefore, the method cannot be reliably used without validating the settings for each experimental condition by measuring the amount and kinetics of inactivation at the prepulse potential [23]. But even if these experimental requirements are met, the prepulse-inactivation-subtraction method yields limited information on other inactivating and non-inactivating current components, which may be remodeled as part of the experiment. Therefore, this isolation method is not very well suited to compare the different genotypes of an I_to_-related transgenic mouse model.

As an alternative method many investigators apply long depolarizing pulses to activate all components of the outward current and let them inactivate one by one over many seconds [24–26]. Then, based on multi-exponential fitting of the current decay, multiple time constants and their amplitudes are obtained, and the individual decay components can be assigned to individual current components [24–26]. Of course, this method depends critically on the length of the depolarizing pulse and on the number of exponential terms deployed. Based on double-exponential fitting of the compound outward currents obtained with 4.5 s pulses, the terms I_to,f_ (fast transient outward current, first decay component in the majority of cells), I_to,s_ (slow transient outward current, first decay component in a subset of cells), I_K,slow_ (slowly inactivating current, second decay component) were coined by Guo et al. [24] and Xu et al. [26], in addition to the non-inactivating steady-state current component I_SS_. Triple-exponential fitting was utilized by these authors only to describe the current decay kinetics in cells from left ventricular septum (but not apex) to account for the putative presence of both I_to,f_ and I_to,s_ [24, 26]. By contrast, Liu and coworkers used rigorous triple-exponential fitting to describe the decay kinetics of currents obtained with pulse lengths between 5 and 25 s from all ventricular myocytes [25]. These authors state that I_to,f_ and I_to,s_ cannot be kinetically separated based on macroscopic inactivation, and thus, the first of three decay components represents either I_to,f_ or I_to,s_ or a mixture of the two [25]. For the second and third decay component the terms I_K,slow1_ and I_K,slow2_, respectively, were coined [25]. As an unbiased way to find out which of the two fitting methods is better suited to describe our data, we initially applied them in parallel to male KChIP2^+/+^ myocytes and compared the results with the ones obtained by the prepulse-inactivation-subtraction method (see Fig 1). Due to significantly lower RMS values and the perfect match between τs and τ1 and between As and A1 we decided to use triple-exponential fitting for all myocytes of the present study if appropriate. Therefore, we like to adhere to the classification and nomenclature of inactivating current components used by Liu and coworkers (τ1 and A1: I_to_; τ2 and A2: I_K,slow1_; τ3 and A3: I_K,slow2_; [25]). In our study double-exponential fitting was only appropriate for the currents obtained from a small fraction of KChIP2^-/-^ myocytes, suggesting the loss of one current component (see below).

Due to peak amplitudes of ~ 10 nA (Tables 1 and 2) it cannot be excluded that the currents obtained with our whole-cell patch-clamp recordings are to some degree distorted by insufficient series resistance compensation (see Materials and Methods). Also, I_K1_-mediated tail currents were often seen upon repolarization, indicative of K^+^ accumulation in t-tubules and consequently a shift in E_K_ ([29]; see for instance Fig 3). Despite these potential sources of error, our triple-exponential fit results for wild-type myocytes are in very good agreement with the ones obtained based on the same analysis method by Liu et al. using 5 s pulses (mean I_to_ time constant ~ 55 ms; [25]) and by Costantini et al. using 3.5 s pulses (mean I_to_ density ~ 18 pA/pF; [30]). Liu and coworkers found out that maximal reliability of the triple-exponential fit is reached with pulse lengths greater than 20 s [25]. With 25 s pulses to +60 mV their mean I_to_ inactivation time constant was 54 ms and their mean I_to_ density 24 pA/pF, still very close to our results (see Tables 1 and 2). Our I_K,slow1_ and I_K,slow2_ time constants may be understimated due to the shorter pulse length of 5 s used in the present study, whereas the on average smaller current densities may be explained by our test potential of +40 mV instead of +60 mV [25, 30].

In the present study multi-exponential fitting was not applied to the test pulse currents obtained with the double-pulse protocol and the variable prepulse protocol to study recovery from inactivation and the voltage dependence of inactivation, respectively. Rather, these inactivation properties were cumulatively determined for the compound outward current by processing the relative peak amplitudes of the test pulse currents. To account for the presence of current components with different recovery kinetics and different voltage dependences of inactivation the data were fitted with a double-exponential function (recovery from inactivation) and the sum of two Boltzmann-functions (voltage dependence of inactivation). At the expense of this simplification we were able to acquire data from a large number of cells with minimal SEM, a favorable condition to perform ANOVA over multiple groups. Aware that either recovery component and either component of the voltage dependence of inactivation may reflect more than one current component, we assumed that I_to_ properties were merely represented by the faster recovery component (τ_rec_1), at least for KChIP2^+/+^. Our values for τ_rec_1 (see Tables 1 and 2) are larger than most values obtained for the isolated I_to_ component (recovery time constants between 25 and 50 ms; [23–26, 31–33]). We cannot exclude that our τ_rec_1 values are influenced by the fast I_K,slow1_ recovery component and/or I_K,slow2_ recovery kinetics ([25]; see below). On the other hand, τ_rec_1 values cumulatively obtained by Tozakidou et al. (51 ms; [27]) and by Wang & Duff (41 ms; [34]) for the compound outward current, as in the present study, are virtually identical to the I_to,f_ recovery time constant determined by Foeger et al. (51 ms; [31]), which warrants our simplified approach. A further assumption of the present study was that I_to_ properties are represented by the more negative component of the voltage dependence of inactivation (V_1/2_1). After all, V_1/2_1, rather than V_1/2_2, was modified in a KChIP2 genotype-dependent manner (see below). But appart from that, the assumption is directly related to the -40 mV prepulse used to completely inactivate I_to_, meaning that the corresponding V_1/2_ value must be more negative than that. In fact, our V_1/2_1 values (see Tables 1 and 2) are very similar to the one obtained by Brouiette et al. (-56 mV, [23]) for the I_to_ component isolated with the prepulse-inactivation-subtraction method.

### Remodeling in I_to_-related transgenics

There are now good protein candidates for the molecular correlates of individual inactivating current components in murine left ventricular myocytes [1, 35]: I_to,f_ is thought to be mainly carried by Kv4.2 channels associated with KChIP2 (fast recovery from inactivation), and I_to,s_ by Kv1.4 channels (slow recovery from inactivation). Both Kv4.2/KChIP2 and Kv1.4 channels may potentially contribute to I_to_, although in most wild-type cardiomyocytes Kv4.2/KChIP2-mediated I_to,f_ governs I_to_ (see below). I_K,slow1_ is thought to be carried by Kv1.5 channels and I_K,slow2_ by Kv2.1 channels. In order to unveil the molecular correlate of the I_to_ component(s) in murine cardiomyocytes, different transgenic mouse models were deployed. Initially, it was found that targeted deletion of the Kv1.4 gene (Kv1.4^-/-^) left I_to_ virtually unaffected [36], showing that I_to_ consists mainly of I_to,f_ for which Kv1.4 is an unlikely candidate. By contrast, I_to_ was strongly reduced by tissue-specific overexpression of a dominant-negative Kv4.2 pore mutant (Kv4.2W362F; [3, 24]) or N-terminal fragment (Kv4.2S1-S4; [37]), showing that the I_to,f_ component is carried by members of the Kv4 subfamily. Finally, targeted deletion of the Kv4.2 gene (Kv4.2^-/-^; [38]) and the Kv4.3 gene (Kv4.3^-/-^; [33]), respectively, demonstrated that I_to,f_ is carried mainly by Kv4.2 rather than Kv4.3 in mice. Notably, it has been recognized early on that dominant-negative Kv4.2W362F expression causes remodeling processes in the form of I_to,s_ up-regulation [3, 39], which was not seen in cardiomyocytes from mice overexpressing Kv4.2W362F in a Kv1.4^-/-^ genetic background (Kv4.2W362FXKv1.4^-/-^; [39]). These results led to the conclusion that Kv1.4 channels are responsible for I_to,s_ up-regulation. An emergence of I_to,s_ was also observed in Kv4.2^-/-^ myocytes, however, specific deletion of the Kv4.2 gene may allow the up-regulation of a Kv4.3-mediated I_to,f_ [32].

Targeted deletion of the KChIP2 gene (KChIP2^-/-^; [9, 21, 31, 40]) has been used to study the role of KChIP2 in I_to_ expression, the present study included. Initially, Kuo and coworkers reported a complete loss of I_to_ for the homozygous KChIP2 knockout (KChIP2^-/-^), although, based on the results of the prepulse-inactivation-subtraction method, a not further quantified "background level" of I_to_ was apparently left in the KChIP2^-/-^ myocytes [9]. This is very similar to our results, when using the prepulse-inactivation-subtraction method (see S1 Fig and S1 Table). However, a clear separation of two different KChIP2^-/-^ myocyte populations, one with a complete loss of I_to_ and one with a kinetically modified I_to_, may only be possible by multi-exponential fitting. Analogous to our multi-exponential fitting strategy, Foeger et al. deployed the same number of exponential terms to describe the compound outward current decay kinetics in KChIP2^+/+^ and KChIP2^-/-^ myocytes [31]. Both the results of prepulse-inactivation-subtraction and the results of multi-exponential fitting can be interpreted as an up-regulation of a novel (or previously hidden) current component in KChIP2^-/-^ myocytes in the absence of Kv4.2 channel-mediated I_to,f_. The results are comparable to the ones obtained with dominant-negative Kv4.2W362F expression [3, 24] or with the Kv4.2^-/-^ deletion mutant [38] and may be explained by I_to,s_ up-regulation [31]. Notably, for a minor fraction of KChIP2^-/-^ myocytes we reduced the number of exponential terms (double-exponential instead of triple-exponential) in order to obtain decent fit results. It is intriguing that double-exponential analysis of the currents obtained from these KChIP2^-/-^ myocytes apparently yielded the I_K,slow1_ and I_K,slow2_ components. We think that only these myocytes underwent a complete loss of I_to_, meaning that I_to,f_ was eliminated and I_to,s_ not up-regulated, reminiscent of the current kinetics found in Kv4.2W362FXKv1.4^-/-^ myocytes [39].

Deviding the (novel) I_to,s_ density by the (native) I_to,f_ density represents a possibility to quantify I_to_ remodeling for a given experimental approach. Notably, this rough estimate yields relatively uniform results when applied to various published data: Foeger et al. found a fraction of 0.421 for myocytes of KChIP2^-/-^ left ventricular apex [31], meaning that after the complete loss of I_to,f_, up-regulation of the novel current component restores 42.1% of the native I_to,f_ density. Barry et al. found a fraction of 41.3% for myocytes expressing Kv4.2W362F [3] and Guo et al. a fraction of 38.4% in Kv4.2^-/-^ myocytes [38]. Intriguingly, even in naive myocytes devoid of I_to,f_ the relative I_to,s_ fraction, compared to I_to,f_-expressing cells, is 43.5% [26]. In the present study, deviding D1 of KChIP2^-/-^ myocytes by D1 of KChIP2^+/+^ myocytes (see Tables 1 and 2) as an estimate of the I_to,s_/I_to,f_ fraction yielded 31.4% for male and 36.3% for female myocytes. These values are a little lower than the one obtained by Foeger et al. for KChIP2^-/-^ myocytes [31] but correlate extremely well with the fraction of 31.6% obtained by Liu et al. for Kv4.2^-/-^ myocytes [32]. A relatively uniform I_to,s_/I_to,f_ fraction (between 30 and 40%) supports the notion that a common and tightly regulated molecular mechanism underlies the observed remodeling. In accordance with Kv1.4 up-regulation as the basis for the I_to,s_ emergence when Kv4.2/KChIP2-mediated I_to,f_ is absent [39] Thomsen et al. showed that in KChIP2^-/-^ myocytes a heteropodatoxin-sensitive current component was absent and the 4-aminopyridine-sensitive component was increased [21]. However, their RT-PCR data suggested increased Kv4.2, Kv4.3 and Kv1.5 messages in KChIP2^-/-^ ventricles [21]. By implication, up-regulation of Kv1.5 in KChIP2^-/-^ myocytes would be in accordance with the reported suppression of Kv1.5 on heterologous coexpression with KChIP2 [41]. However, our data do not support I_K,slow1_ up-regulation in KChIP2^-/-^ myocytes of either sex (A2 and D2 in Tables 1 and 2).

The kinetics of recovery from inactivation are usually a good indicator of electrical remodeling. Our results indicate the virtual absence of typical I_to,f_ recovery kinetics (see above) in KChIP2^-/-^ myocytes (see Tables 1 and 2). This probably allows the fast I_K,slow1_ recovery component and/or I_K,slow2_ recovery kinetics (recovery time constants between 350 and 400 ms, [25]) to come to the fore. The second recovery component was also slowed in KChIP2^-/-^ myocytes, probably due to both a shift of I_K,slow_ recovery components into the first component and an up-regulation of the I_to,s_ recovery component (recovery time constant ~ 2000 ms, [25]; see Tables 1 and 2). An intriguing finding of the present study is the modified voltage dependence of inactivation found in KChIP2^-/-^ myocytes. We observed that the more negative component (V_1/2_1) was shifted to even more negative potentials but only accounted for 10–12% (Tables 1 and 2). By implication, a causal relationship between the absence of KChIP2 and the negative shift in V_1/2_1 is supported by the fact that heterologous KChIP2 coexpression shifts the voltage dependence of Kv4 channel inactivation in the positive direction [14]. Thus, the modified inactivation curves of KChIP2^-/-^ myocytes may indicate a small amount of Kv4 channels reaching the cardiomyocyte plasma membrane without the need of KChIP2. This hypothesis is supported by our relA1 values (16% in male and 20% in female KChIP2^-/-^ myocytes; Tables 1 and 2), but not by Foeger et al., who reported the absence of Kv4.2 protein in KChIP2^-/-^ ventricles [31]. A possible coexistence of I_to,s_ and I_to,f_ in the absence of KChIP2 needs further investigation, considering prominent Kv4.3 up-regulation [21, 32] as a candidate mechanism.

### Dynamics of functional Kv4.2/KChIP2 expression

Functional expression of Kv4 channels (i.e., subunit assembly, trafficking to the surface membrane and complex stability) is known to be augmented by KChIPs in heterologous expression systems [8, 14, 16–19], but the role of KChIPs in cardiomyocytes has remained undefined for a long time. In murine ventricular myocytes I_to_ is mainly carried by Kv4.2 channels associated with KChIP2, and targeted deletion of the KChIP2 gene (KChIP2^-/-^) virtually eliminates the Kv4.2 channel-mediated I_to_ [9, 21, 31, 33, 38]. The latter finding alone represents a strong argument for KChIP2 being essential for the functional Kv4.2 expression and thus, being a key regulator of I_to_. The critical role of KChIP2 in I_to_ expression becomes even more evident by the finding that there is a gene dosage effect, with an intermediate I_to_ magnitude in the heterozygous genotype (KChIP2^+/-^), as reported by Kuo et al. [9]. In order to corroborate this finding, we applied a completely different I_to_ isolation method and compared different KChIP2 genotypes in male and female myocytes. We found a distinct KChIP2 gene dosage effect on I_to_ magnitude in both sexes.

In diploid organisms the presence of two alleles for a gene locus is set to 100%. This percentage can be larger, for instance in trisomies (150%), or 50% if one allele is missing, as in the KChIP2^+/-^ genotype. We asked how strictly a given allelic percentage is converted into a certain phenotype (i.e., amount of protein and eventually protein function). The KChIP2 genotype dependence can be quantified by calculating the relative I_to_ magnitude for KChIP2^+/-^ compared to KChIP2^+/+^. In the present study we obtained heterozygous I_to_ fractions of 63% and 66%, respectively, and heterozygous I_to_ density fractions of 71% and 61%, respectively, for male and female myocytes. Our fractions are larger than the density fraction reported by Kuo et al. (42%; [9]), and also larger than the theoretical value of 50%, assuming a strict conversion of the number of alleles. A possible explanation for this deviation would be the up-regulation of a KChIP2-unrelated I_to_ component (e.g., I_to,s_), not only in KChIP2^-/-^ but also in KChIP2^+/-^ myocytes. In that case one would expect a modulation of inactivation properties. However, we detected no significant differences concerning the inactivation properties of KChIP2^+/+^ and KChIP2^+/-^ myocytes, making an appreciable I_to,s_ up-regulation in KChIP2^+/-^ myocytes unlikely. It should be noted that in female KChIP2^+/-^ myocytes a vague KChIP2 gene dosage effect on inactivation properties was seen (see Fig 7A and 7D). In this context it is intriguing that a number of inactivation parameters, all related to I_to_, differed between male and female KChIP2^+/+^ myocytes (see Table 2). Some of the observed differences (faster I_to_ decay kinetics and more negative V_1/2_1) create the impression that the KChIP2 effect on inactivation properties has not become fully manifest in female myocytes. However, this does not apply to the kinetics of recovery from inactivation, because τ_rec_1 was smaller in female than in male myocytes. It has been reported that Kv1.5 and Kv4.3 expression is lower in ventricular myocytes from female mice [28, 42]. Althogh these findings would offer a possible explanation for the different inactivation properties in female myocytes, we detected neither gender-dependent differences in I_K,slow_ densities (in accordance with Brunet et al. [43]) nor a KChIP2 genotype-dependent modulation of I_K,slow_ densities (in contrast to Thomsen et al. [21]). A possible connectivity between the characteristic electrophysiological features and the tentative KChIP2 gene dosage effect on inactivation properties in female myocytes, was not further analyzed.

In the present study the partial KChIP2 gene dosage effect in cardiomyocytes (distinct KChIP2 gene dosage effect on I_to_ magnitude but not on inactivation properties) could be reproduced in a heterologous expression system by injecting a fixed amount of Kv4.2 cRNA with variable amounts of KChIP2 cRNA in *Xenopus* oocytes. The observed increase in the measured current amplitudes with increased amounts of KChIP2 cRNA was expected and most likely due to an increased amount of KChIP2 and increased surface expression of Kv4.2 [19, 44, 45]. Moreover, we show that the KChIP2-dependent gain in current amplitude resembles a logarithmic growth curve, with the typical two portions of steep and shallow increment, respectively (see S3 Fig). It is remarkable that the peak current amplitude of an ion channel correlates so tightly with the cRNA dose of its β-subunit, supporting the notion that KChIP2 is a key regulator of functional Kv4.2 expression. Our results for macroscopic inactivation and recovery from inactivation of Kv4.2 channels in oocytes were similar to the ones obtained by Goltz et al. with KChIP2 [46] and by Kitazawa et al. with KChIP4 [44]. However, the results of the present study provide clear evidence that the KChIP2 dose dependence of Kv4.2 surface expression differs from the KChIP2 dose dependence of Kv4.2 inactivation gating (see Fig 8F–8H and S3 Fig). Two remarkable findings of our oocyte experiments reflect the partial KChIP2 gene dosage effect: First, current amplitude could be further increased, albeit shallowly, by using KChIP2 cRNA amounts well above saturation for effects on macroscopic inactivation and recovery from inactivation; and secondly, varying the KChIP2 cRNA amount in the steep portion of the logarithmic function curve for peak current amplitude has neglible effects on inactivation gating (see Fig 8 and S3 Fig). Above all, it is intriguing that, by using low amounts of KChIP2 cRNA, the I_to_ inactivation properties seen in KChIP2^+/+^ and KChIP2^+/-^ myocytes could be reproduced (see Fig 8B and 8C). Furthermore, when reducing the KChIP2 cRNA amount by 50% (from 0.4 to 0.2 ng per oocyte or from 0.2 to 0.1 ng per oocyte) the fractional amplitude of oocyte currents is 63% or 69%, respectively. The latter results are directly transferable to our KChIP2 gene dosage effect on I_to_ magnitude in cardiomyocytes (heterozygous I_to_ fractions of 63 and 66%; see above). Our combined results support the notion that in cardiomyocytes a KChIP2 allele number of 50% is not strictly translated into I_to_ magnitude. They imply that the wild-type allele number (100%) provides saturating amounts of KChIP2 to ensure I_to_ expression.

Increasing the KChIP2 cRNA amount well above a value that can reproduce our cardiomyocyte results (e.g., 0.8 ng per oocyte or higher) caused a further slowing of macroscopic inactivation and further acceleration of recovery from inactivation (see Fig 8). Notably, the KChIP2 dose dependence of τ_inact_1 was smooth and probably reflects the functional stoichiometry of N-type inactivation ([47, 48]; see also S3B Fig). By contrast, τ_inact_2 and τ_rec_ showed distinct levels, probably reflecting the fact that both τ_inact_2 and τ_rec_ are merely related to the allosteric mechanism of Kv4.2 closed-state inactivation ([49]; see also S3C and S3D Fig). Our oocyte data raise the question whether in KChIP2^+/+^ myocytes the KChIP2 effects on inactivation properties are at all saturated, which automatically leads to the question regarding the stoichiometry of Kv4.2/KChIP2 complexes in the cardiomyocyte membrane (i.e., how many KChIP2 molecules are bound to a Kv4.2 channel). Based on the analysis of purified channels and crystal structure data it has been commonly thought that Kv4 α-subunits and KChIPs form an octameric complex with a 4:4 stoichiometry [50–53]. However, in these experimental approaches KChIP was present in excess. More recently, Kitazawa and coworkers found that both the biophysical properties and the stoichiometry of Kv4.2/KChIP4 channels depend critically on the amount of KChIP4 coexpressed in *Xenopus* oocytes [44]. The authors concluded that Kv4.2/KChIP4 channel complexes may have variable stoichiometries depending on the amount of KChIP4 available, and that stoichiometry dictates channel gating. Both Kv4 and KChIP expression are developmentally regulated [54–57], such that their relative amounts and the Kv4/KChIP stoichiometry and, thus I_to_ properties, may change with age. Kv4 and KChIP expression are even regulated based on a circadian rhythm [58, 59], which may allow a more short-term regulation of I_to_ properties. Whether KChIP molecules freely moving in the cytosol [19, 44] are available for spontaneous binding to Kv4.2 channels in the plasma membrane, thereby allowing a most acute form of I_to_ regulation, remains to be examined.

In summary, our results support the notion that cardiomyocytes normally produce enough KChIP2 to ensure I_to_ expression. On the other hand, the molecular correlate of I_to_ seems to be highly dynamic, such that changes in the amount of KChIP2 may create a considerable scope concerning I_to_ properties in remodeling processes including cardiac memory, hypertrophy and heart failure [60].

## Supporting information

S1 Fig Prepulse-inactivation-subtraction method applied to male cardiomyocytes with different KChIP2 genotypes. Individual myocytes were isolated from the left ventricular free wall of male mice and currents were measured with the whole-cell patch-clamp technique. The voltage protocol used for the prepulse-inactivation-subtraction method is depicted on the lower right (D). Compound outward currents (1) were activated by a voltage pulse from -80 to +40 mV. Sodium currents (not recorded at their full size) were inactivated by a brief (8 ms) prepulse to -50 mV. A fraction of outward current was inactivated by a 160 ms prepulse to -40 mV (2). Subtraction of 1–2 yielded a rapidly decaying current trace referred to as I_to_. The decay of this current trace was fitted by a single-exponential function (red). Application of this method is shown for a KChIP2^+/+^ (A) and a KChIP2^+/-^ myocyte (B) and two KChIP2^-/-^ myocytes (C and D). In some KChIP2^-/-^ myocytes the method yielded current traces with extremely small amplitudes (i.e., I_to_ was virtually lost), however, the time course of current decay could still be fitted by a single-exponential function (C).
(PDF)

S2 Fig Expression profiles of Kv4.2 and Kv4.2/KChIP2 channels in *Xenopus* oocytes. Oocytes were surgically removed and treated with collagenase. Predefined amounts of Kv4.2 and KChIP2 cRNA were injected into individual oocytes, and currents were measured with the two-electrode voltage-clamp technique. A. Functional expression of Kv4.2 and Kv4.2/KChIP2 channels over time. Oocytes were injected with 1 ng Kv4.2 cRNA alone or coinjected with 1 ng Kv4.2 and 1 ng KChIP2 cRNA. Peak current amplitudes were measured 24, 48, 72 and 96 h after cRNA injection. B. For each time of recording the fold gain in peak current amplitude in the presence of KChIP2 compared to Kv4.2 alone was determined.
(PDF)

S3 Fig Dependence of Kv4.2 properties on the amount of coinjected KChIP2 cRNA. A fixed amount of 1 ng Kv4.2 cRNA was coinjected with different amounts of KChIP2 cRNA (0, 0.1, 0.2, 0.4, 0.8, 1.6, 3.2, 6.4 and 12.8 ng) into individual oocytes, and currents were recorded 48 h after cRNA injection. Various current parameters were analyzed, and the results were plotted against the amount of coinjected KChIP2 cRNA. A. Peak current amplitude; a logarithmic fit represents a good description of the data, indicating that a steeper portion of the function (low to intermediate KChIP2 cRNA amounts, y < 19 μA) can be distinguished from a shallower portion (high KChIP2 cRNA amounts, y > 19 μA). The KChIP2 cRNA amounts of 0.4 and 0.2 ng per oocyte lie within the steep portion and the relative difference in amplitude is 63%, very similar to the relative differences between the I_to_ amplitudes obtained for KChIP2^+/+^ and KChIP2^+/-^, respectively, in both male (63%) and female cardiomyocytes (66%). B. First inactivation time constant (τ_inact_1) obtained with a double-exponential fit to the current decay; a logarithmic function describes the data well for low to intermediate KChIP2 cRNA amounts (y ≤ 56 ms). However, at higher KChIP2 cRNA amounts τ_inact_1 saturates at ~ 80 ms. C. Second inactivation time constant (τ_inact_2) obtained with a double-exponential fit to the current decay; a logarithmic function does not describe the data very well. The mean τ_inact_2 is ~ 200 ms in the absence of KChIP2, between 400 and 600 ms at low to intermediate amounts and ~ 1000 ms at high amounts of KChIP2 cRNA. D. Recovery time constant (τ_rec_). Similar to τ_inact_2, the recovery data cannot be well described by a logarithmic function. The mean τ_rec_ is ~ 700 ms in the absence of KChIP2, between 150 and 250 ms at low to intermediate KChIP2 cRNA amounts and ~ 40 ms at KChIP2 cRNA amounts of 0.8 ng per oocyte and higher.
(PDF)

S1 Table Data summary for the prepulse-inactivation-subtraction method applied to male cardiomyocytes. (PDF)
