# Supplementary figures and images for: KChIP2 genotype dependence of transient outward current (Ito) properties in cardiomyocytes isolated from male and female mice
Source: PLoS One. 2017 Jan 31;12(1):e0171213. doi: 10.1371/journal.pone.0171213 (PMC5283746; doi:10.1371/journal.pone.0171213)

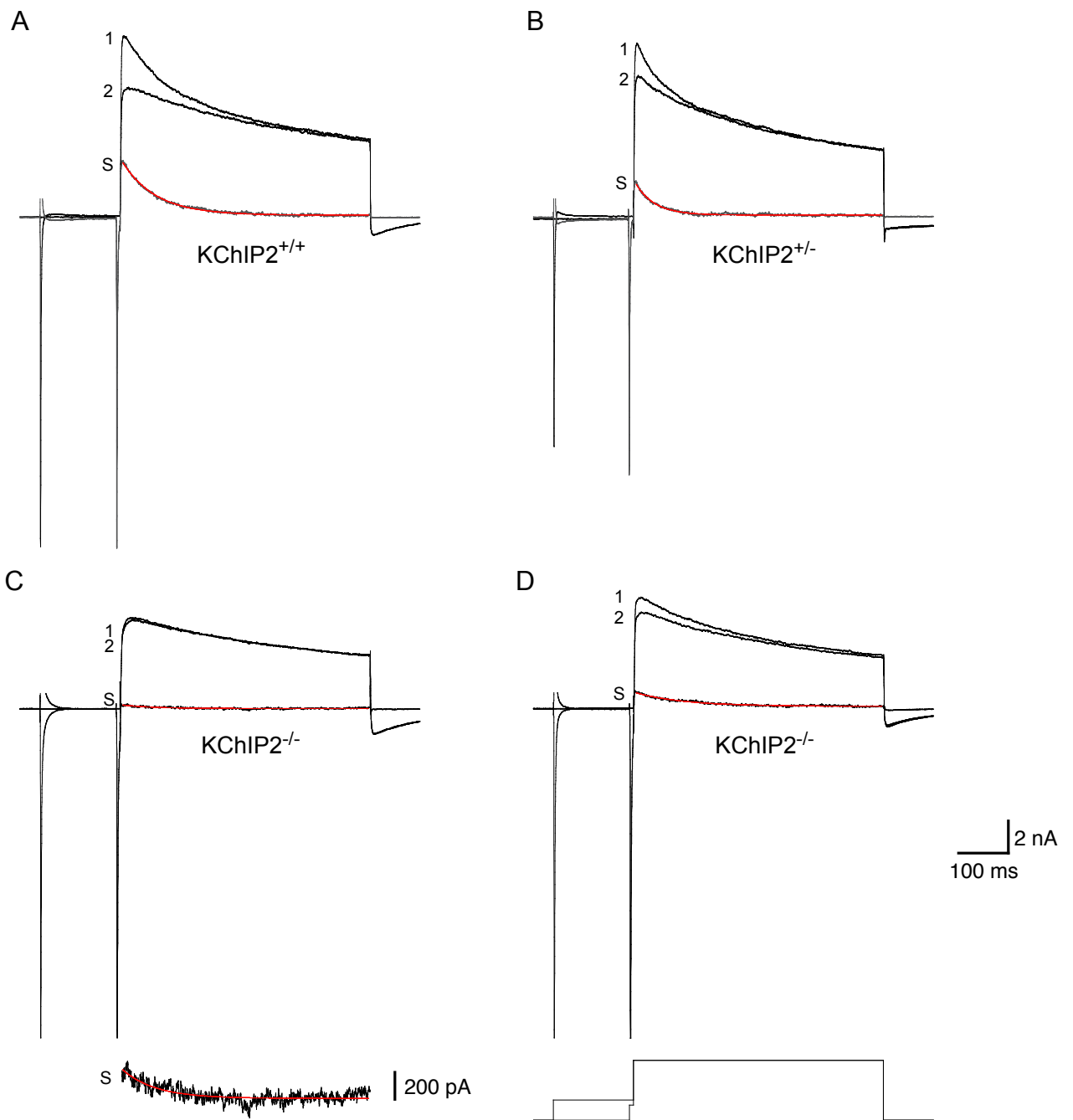

Supplement: S1 Fig — Individual myocytes were isolated from the left ventricular free wall of male mice and currents were measured with the whole-cell patch-clamp technique. The voltage protocol used for the prepulse-inactivation-subtraction method is depicted on the lower right (D). Compound outward currents (1) were activated by a voltage pulse from -80 to +40 mV. Sodium currents (not recorded at their full size) were inactivated by a brief (8 ms) prepulse to -50 mV. A fraction of outward current was inactivated by a 160 ms prepulse to -40 mV (2). Subtraction of 1–2 yielded a rapidly decaying current trace referred to as Ito. The decay of this current trace was fitted by a single-exponential function (red). Application of this method is shown for a KChIP2+/+ (A) and a KChIP2+/- myocyte (B) and two KChIP2-/- myocytes (C and D). In some KChIP2-/- myocytes the method yielded current traces with extremely small amplitudes (i.e., Ito was virtually lost), however, the time course of current decay could still be fitted by a single-exponential function (C). (PDF) [file pone.0171213.s001.pdf]

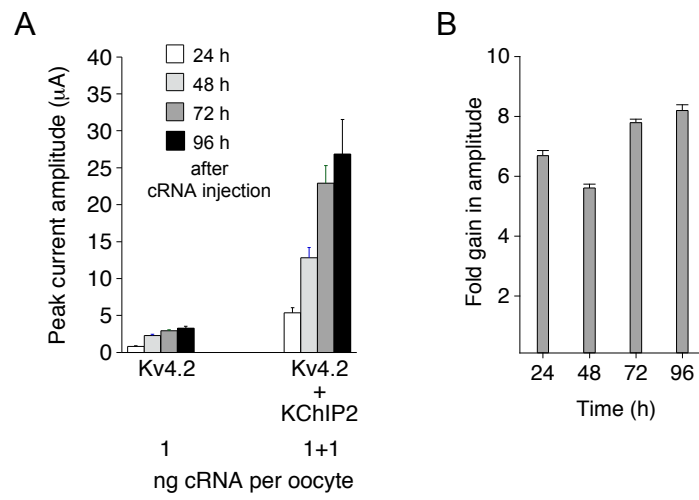

Supplement: S2 Fig — Oocytes were surgically removed and treated with collagenase. Predefined amounts of Kv4.2 and KChIP2 cRNA were injected into individual oocytes, and currents were measured with the two-electrode voltage-clamp technique. A. Functional expression of Kv4.2 and Kv4.2/KChIP2 channels over time. Oocytes were injected with 1 ng Kv4.2 cRNA alone or coinjected with 1 ng Kv4.2 and 1 ng KChIP2 cRNA. Peak current amplitudes were measured 24, 48, 72 and 96 h after cRNA injection. B. For each time of recording the fold gain in peak current amplitude in the presence of KChIP2 compared to Kv4.2 alone was determined. (PDF) [file pone.0171213.s002.pdf]

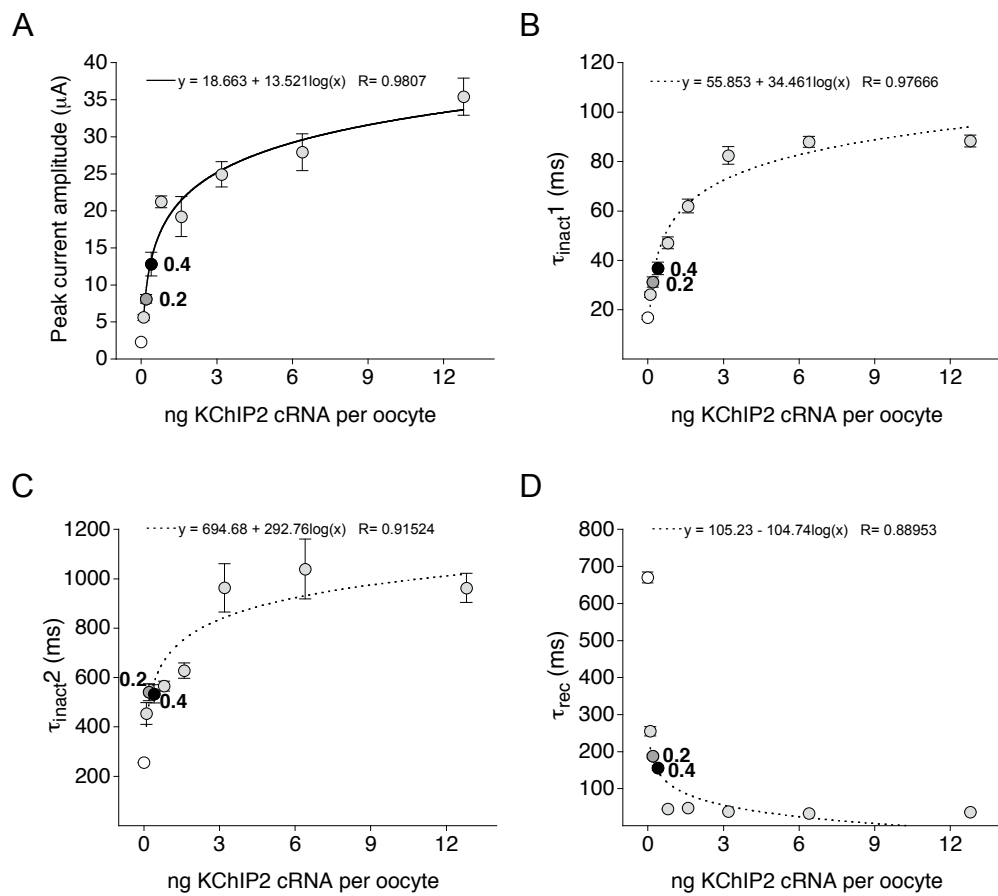

Supplement: S3 Fig — A fixed amount of 1 ng Kv4.2 cRNA was coinjected with different amounts of KChIP2 cRNA (0, 0.1, 0.2, 0.4, 0.8, 1.6, 3.2, 6.4 and 12.8 ng) into individual oocytes, and currents were recorded 48 h after cRNA injection. Various current parameters were analyzed, and the results were plotted against the amount of coinjected KChIP2 cRNA. A. Peak current amplitude; a logarithmic fit represents a good description of the data, indicating that a steeper portion of the function (low to intermediate KChIP2 cRNA amounts, y < 19 μA) can be distinguished from a shallower portion (high KChIP2 cRNA amounts, y > 19 μA). The KChIP2 cRNA amounts of 0.4 and 0.2 ng per oocyte lie within the steep portion and the relative difference in amplitude is 63%, very similar to the relative differences between the Ito amplitudes obtained for KChIP2+/+ and KChIP2+/-, respectively, in both male (63%) and female cardiomyocytes (66%). B. First inactivation time constant (τinact1) obtained with a double-exponential fit to the current decay; a logarithmic function describes the data well for low to intermediate KChIP2 cRNA amounts (y ≤ 56 ms). However, at higher KChIP2 cRNA amounts τinact1 saturates at ~ 80 ms. C. Second inactivation time constant (τinact2) obtained with a double-exponential fit to the current decay; a logarithmic function does not describe the data very well. The mean τinact2 is ~ 200 ms in the absence of KChIP2, between 400 and 600 ms at low to intermediate amounts and ~ 1000 ms at high amounts of KChIP2 cRNA. D. Recovery time constant (τrec). Similar to τinact2, the recovery data cannot be well described by a logarithmic function. The mean τrec is ~ 700 ms in the absence of KChIP2, between 150 and 250 ms at low to intermediate KChIP2 cRNA amounts and ~ 40 ms at KChIP2 cRNA amounts of 0.8 ng per oocyte and higher. (PDF) [file pone.0171213.s003.pdf]
