# Supplementary material for: KChIP2 genotype dependence of transient outward current (Ito) properties in cardiomyocytes isolated from male and female mice
Source: PLoS One. 2017 Jan 31;12(1):e0171213. doi: 10.1371/journal.pone.0171213 (PMC5283746; doi:10.1371/journal.pone.0171213)
Supplement: S1 Table — (PDF) [file pone.0171213.s004.pdf]

S1 Table: **Data summary for the prepulse-inactivation-subtraction method applied to male cardiomyocytes.**

♂	$KChIP2^{+/+}$	$KChIP2^{+/-}$	$KChIP2^{-/-}$
**Current kinetics and magnitudes**			
τs (ms)	62.2 ± 2.1	54.0 ± 2.6	203 ± 75 **
As (nA)	3.11 ± 0.30	2.35 ± 0.19 *	0.61 ± 0.05 **
Cap (pF)	189 ± 12	173 ± 8	172 ± 11
Ds (pA/pF)	17.1 ± 1.9	14.2 ± 1.1	3.8 ± 0.3 **
	(n = 12)	(n = 36)	(n = 25)

Analysis results obtained for the I_{to} kinetics and magnitudes for male myocytes with different KChIP2 genotypes. * significantly different from $KChIP2^{+/+}$; ** significantly different from both $KChIP2^{+/+}$ and $KChIP2^{+/-}$ (one way ANOVA); abbreviations are explained in the text.
